# Mitochondrial antiviral signaling protein enhances MASLD progression through the ERK/TNFα/NFκβ pathway

**DOI:** 10.1097/HEP.0000000000000930

**Published:** 2024-05-16

**Authors:** Eva Nóvoa, Natália da Silva Lima, Maria J. Gonzalez-Rellan, Maria D.P. Chantada-Vazquez, Joanne Verheij, Amaia Rodriguez, Eva M. Esquinas-Roman, Marcos F. Fondevila, Mirja Koning, Uxia Fernandez, Alba Cabaleiro, Tamara Parracho, Jose Iglesias-Moure, Samuel Seoane, Begoña Porteiro, Adriana Escudero, Ana Senra, Roman Perez-Fernandez, Miguel López, Miguel Fidalgo, Diana Guallar, Maria L. Martinez-Chantar, Carlos Dieguez, Marta Varela-Rey, Vincent Prevot, Markus Schwaninger, Abraham Meijnikman, Susana B. Bravo, Gema Frühbeck, Ruben Nogueiras

**Affiliations:** 1 Department of Physiology, CIMUS, University of Santiago de Compostela, Santiago de Compostela, Spain; 2 CIBER Fisiopatologia de la Obesidad y Nutrición (CIBERobn), A Coruña, Spain; 3 Proteomic Unit, Health Research Institute of Santiago de Compostela (IDIS), Santiago de Compostela, A Coruña, Spain; 4 Department of Pathology, Amsterdam University Medical Center, Amsterdam, The Netherlands; 5 Department of Endocrinology & Nutrition, Metabolic Research Laboratory, Clínica Universidad de Navarra, University of Navarra, IdiSNA, Navarra, Spain; 6 Gene Regulatory Control in Disease Laboratory, Center for Research in Molecular Medicine and Chronic Diseases (CIMUS), Instituto de Investigación Sanitaria de Santiago de Compostela (IDIS), University of Santiago de Compostela, Santiago de Compostela, A Coruña, Spain; 7 Liver Disease Lab, BRTA CIC bioGUNE, Centro de Investigación Biomédica en Red de Enfermedades Hepáticas y Digestivas (CIBERehd), Derio, Bizkaia, Spain; 8 Univ. Lille, Inserm, CHU Lille, Laboratory of Development and Plasticity of the Neuroendocrine Brain, Lille Neuroscience & Cognition, UMR-S 1172, European Genomic Institute for Diabetes (EGID), Lille, France; 9 Institute for Experimental and Clinical Pharmacology and Toxicology, University of Lübeck, Lübeck, Germany; 10 Department of Internal and Experimental Vascular Medicine, Amsterdam University Medical Centers, Location AMC, Amsterdam, The Netherlands; 11 Galician Agency of Innovation (GAIN), Xunta de Galicia, Santiago de Compostela, Spain

## Abstract

**Background and Aims:**

Mitochondrial antiviral signaling protein (MAVS) is a critical regulator that activates the host’s innate immunity against RNA viruses, and its signaling pathway has been linked to the secretion of proinflammatory cytokines. However, the actions of MAVS on inflammatory pathways during the development of metabolic dysfunction–associated steatotic liver disease (MASLD) have been little studied.

**Approach and Results:**

Liver proteomic analysis of mice with genetically manipulated hepatic p63, a transcription factor that induces liver steatosis, revealed MAVS as a target downstream of p63. MAVS was thus further evaluated in liver samples from patients and in animal models with MASLD. Genetic inhibition of MAVS was performed in hepatocyte cell lines, primary hepatocytes, spheroids, and mice. MAVS expression is induced in the liver of both animal models and people with MASLD as compared with those without liver disease. Using genetic knockdown of MAVS in adult mice ameliorates diet-induced MASLD. In vitro, silencing MAVS blunts oleic and palmitic acid–induced lipid content, while its overexpression increases the lipid load in hepatocytes. Inhibiting hepatic MAVS reduces circulating levels of the proinflammatory cytokine TNFα and the hepatic expression of both TNFα and NFκβ. Moreover, the inhibition of ERK abolished the activation of TNFα induced by MAVS. The posttranslational modification *O*-GlcNAcylation of MAVS is required to activate inflammation and to promote the high lipid content in hepatocytes.

**Conclusions:**

MAVS is involved in the development of steatosis, and its inhibition in previously damaged hepatocytes can ameliorate MASLD.

## Graphical abstract

**Figure:**
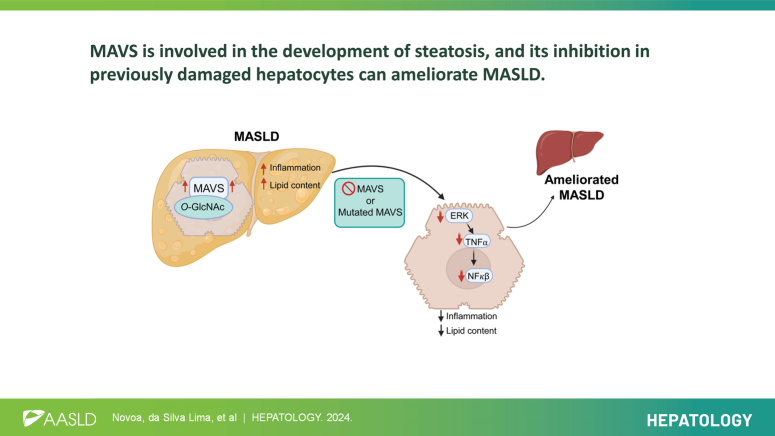

## INTRODUCTION

Metabolic dysfunction–associated steatotic liver disease (MASLD) is a metabolic disorder marked by hepatic steatosis and at least 1 cardiometabolic risk factor, progressing from simple steatosis to steatohepatitis, fibrosis, cirrhosis, and HCC.^1^ Its pathogenesis involves genetic, dietary, microbiota, and inflammatory factors. MASLD affects 25% of the global population, driven by obesity and sedentary lifestyles, making it a major cause of chronic liver disease.^2^ Understanding the multiple and complex molecular pathways implicated in MASLD onset and progression is a major priority.

One of the numerous molecules reported to be involved in this disease is the transcription factor p63, which is in the family comprising p53, p63, and p73.^3^ The TAp63 isoform, which includes the transactivation domain, is elevated in the liver of animal models and patients with obesity and MASLD.^4^ TAp63-induced hepatic fat content is mediated by the activation of IKKβ and endoplasmic reticulum stress.^4^ Of note, inflammation is one of the hallmarks of metabolic dysfunction-associated steatohepatitis (MASH).^5^ The prevailing notion is that abnormal fatty acid accumulation in hepatocytes is considered the “first hit” in MASLD, followed by multiple hits that contribute in parallel to disease progression.^6^ One of these hits is inflammation, as proinflammatory cytokines damage hepatocytes that are sensitized by their organelle’s stress in MASLD, although the mechanisms by which the different hits lead to MASLD remain largely unknown.

Mitochondrial dysfunction also plays a pivotal role in the pathogenesis of MASLD, contributing to oxidative stress, impaired lipid metabolism, and inflammation within hepatocytes. Dysfunctional mitochondria exacerbate the proinflammatory milieu in MASLD and activation of inflammatory signaling pathways, such as NF-κβ and inflammasome activation. Mitochondrial antiviral-signaling protein (MAVS) is localized mainly in the mitochondrial outer membrane, although it has also been detected in peroxisome and mitochondrial-associated endoplasmic reticulum membranes.^7,8^ MAVS (also known as virus-induced signaling adaptor or IFNβ promoter stimulator protein-1) is essential for antiviral innate immunity.^9^ Upon viral recognition, cytosolic proteins such as retinoic acid-inducible gene I and melanoma differentiation–associated protein 5 activate MAVS, initiating an immune response through interferon regulatory factors 3 and 7 (IRF3/7) and NFκβ transcription factors. This leads to the expression and secretion of proinflammatory cytokines and antiviral genes.^9,10^ As activation of MAVS signaling upon RNA virus infection also leads to an increase of glucose metabolic pathways, MAVS is considered a key factor in linking glucose metabolism to antiviral innate immunity.^11^ Interestingly, *O*-GlcNAcylation is a highly regulated, reversible posttranslational modification involved in a wide variety of metabolic signals and cellular processes,^12,13^ and this modification has been reported to be essential for the host antiviral innate immunity of MAVS.^11,14^

In this work, we used unbiased proteomics to analyze the liver of mice with manipulated TAp63 levels. We identified that MAVS is regulated by TAp63: it is upregulated after TAp63 activation and downregulated after p63α inhibition. MAVS activation was consistent across various MASLD animal models and in human MASLD liver samples. In both in vitro and in vivo settings, MAVS overexpression increased hepatocyte lipid accumulation, while MAVS inhibition relieved TAp63- and diet-induced steatosis. These effects on lipid metabolism were mediated through the ERK/TNFα/NFκβ pathway, as ERK, TNFα, or NFκβ inhibition attenuated MAVS-induced lipid accumulation. Furthermore, mutating the *O*-GlcNAcylated residue Thr373 on MAVS hindered its effects, underscoring the importance of *O*-GlcNAcylation in MAVS-mediated inflammation and lipid accumulation.

## METHODS

### Animals and diets

Animal protocols were approved by the Committee at the University of Santiago de Compostela and received humane care according to the criteria outlined in the “Guide for the Care and Use of Laboratory Animals.”

### Cohort of patients with NASH for western blot analysis

All reported investigations were carried out in accordance with the principles of the Declaration of Helsinki, as revised in 2013 and approved by the Hospital’s Ethical Committee responsible for research (protocol 2021.005). Written informed consent was obtained from all the participants. Anthropometric, biochemical, and clinical characteristics of patients are shown in Supplemental Table S2, http://links.lww.com/HEP/I457.

For further details, see Supporting Participants and Methods. Lists of primers and antibodies used are shown in Supplemental Tables S3 and S4. Uncropped western blots are shown in Supplemental Figure S10, http://links.lww.com/HEP/I457.

## RESULTS

### MAVS is positively regulated by p63 and increases in diet-induced animal models of MASLD

**FIGURE 1:**
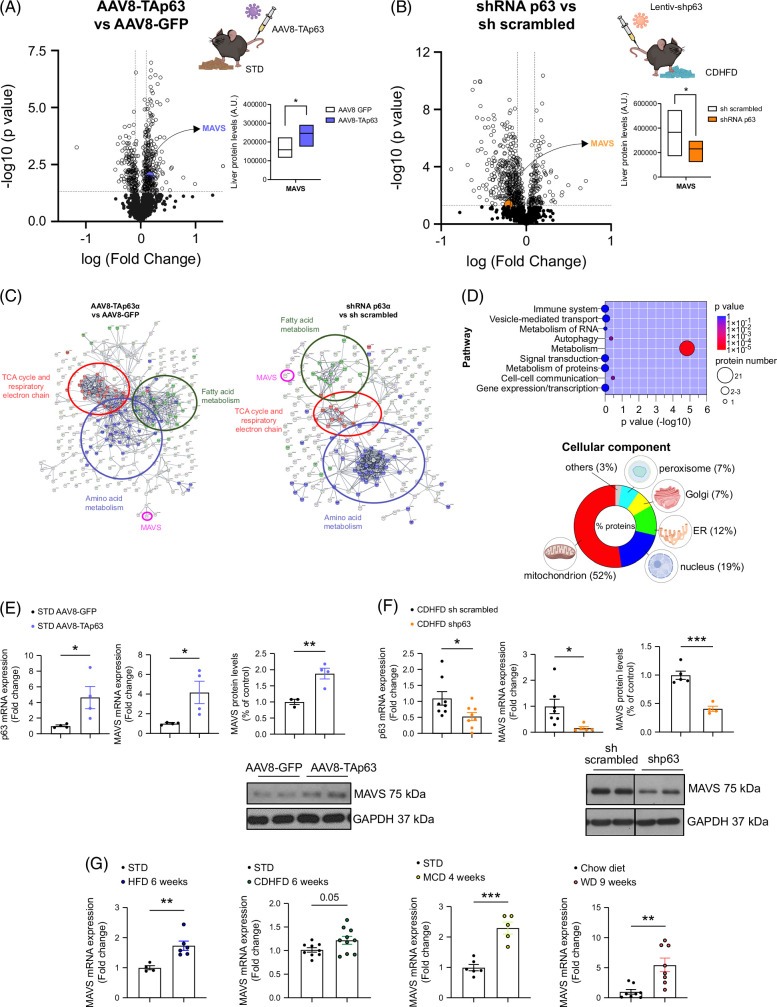
Hepatic MAVS is upregulated in different in vivo and in vitro models of MASLD and MASH. (A, B) Volcano plots of hepatic protein expression determined by LC-MS/MS proteomics of (A) mice fed a standard diet (STD), with TAp63 overexpressed specifically in liver compared to the control group (n = 3 per group), or (B) mice fed a choline-deficient high-fat diet (CDHFD) for 12 weeks, with hepatic p63 downregulated as compared to the control group (n = 3 per group). Values obtained for MAVS are represented in the graph. (C) Protein-protein interaction network of deregulated proteins involved in metabolism according to the STRING database. Node color indicates the 3 main affected functions: TCA and respiratory electron chain (red), fatty acid metabolism (green), and amino acid metabolism (blue). MAVS is indicated in pink. (D) Reactome pathway classification of deregulated proteins in the liver of mice from (A) and (B), showing the number of proteins included in each category and the associated FDR. The same proteins were also classified according to the cellular component using the FunRich tool. The size of the circumference is proportional to the number of proteins deregulated in the metabolic pathway. On the other hand, the color indicates the significance of the *p* value, that is, red is more significant than blue. (E, F) mRNA levels of p63 and MAVS, as well as MAVS protein levels, in mice in the conditions as in (A) and (B) (n = 4–8 per group). (G) MAVS mRNA in the liver of mice fed an STD, a high-fat diet (HFD) (n = 4–6), a choline-deficient plus high-fat diet (CDHFD) (n = 8–10), a methionine-and-choline-deficient (MCD) diet (n = 5–6 per group), and a Western diet (WD) (n = 8). HPRT and GAPDH were used to normalize mRNA and protein levels, respectively. Data are presented as mean ± SEM; ∗*p <* 0.05, ∗∗*p <* 0.01, ∗∗∗*p <* 0.001, Student *t* test. Abbreviations: MASH, metabolic dysfunction-associated steatohepatitis; MASLD, metabolic dysfunction–associated steatotic liver disease; MASH, metabolic dysfunction-associated steatohepatitis; MAVS, mitochondrial antiviral-signaling protein.

Hepatic TAp63 overexpression induces steatosis, while p63 inhibition ameliorates diet-induced steatosis.^4^ Proteomic analyses in these models revealed novel pathways and regulators of hepatic lipid accumulation (Figures 1A–D). Volcano plots show multiple changes in hepatic protein levels upon TAp63 manipulation (Figures 1A, B). A protein interactome network displayed the TCA cycle, respiratory electron chain, fatty acid, and amino acid metabolism alterations after overexpression and knockdown of p63 (Figure 1C). We specifically searched for proteins oppositely expressed in mice following the overexpression or knockdown of p63; we found that p63 positively regulated 43 proteins. Analyzing this protein set revealed a significant overrepresentation (44%) of metabolism-related proteins, with mitochondria identified as the most affected cell component (Figure 1D). Notably, MAVS was induced by TAp63 and downregulated by p63 inhibition, prompting further investigation into its role.

Corroborating our proteomic results, both MAVS mRNA expression and protein levels increased upon TAp63 induction in the liver (Figure 1E), while they were reduced upon hepatic p63α inhibition in diet-induced obese mice (Figure 1F). MAVS expression was assessed in livers from mouse models of diet-induced MASLD and MASH, including mice fed a high-fat diet for 6 weeks, a choline-deficient plus high-fat diet (CDHFD) for 6 weeks, a methionine- and choline-deficient (MCD) diet for 4 weeks, or a Western diet (WD) for 9 weeks. In all 3 animal models, MAVS mRNA was elevated (Figure 1G), suggesting a consistent increase in MAVS expression in preclinical models of MASLD.

### MAVS is increased in the liver of people with MASLD

**FIGURE 2:**
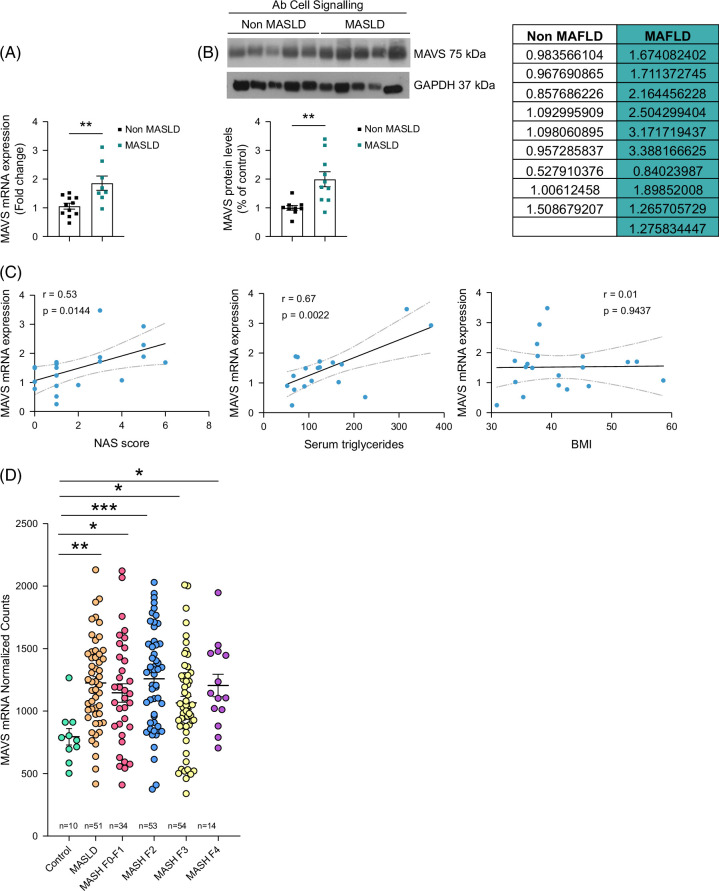
MAVS is increased in the liver of patients with MASLD. (A) MAVS mRNA expression in the liver of patients without MASLD (non-MASLD) (n = 11) or MASLD (n = 8). (B) MAVS protein levels in the liver of patients without MASLD (non-MASLD) (n = 9) or MASLD (n = 10). (C) Correlation between NAS score, serum triglycerides, and BMI with MAVS mRNA levels. (D) Normalized DESeq2 counts for MAVS mRNA expression across the GSE135251 data set composed of liver samples from control patients and patients with 5 grades of MASLD progression. HPRT and GAPDH were used to normalize mRNA and protein levels, respectively. Lines indicate splicing in the same gel. Data are presented as mean ± SEM; ∗∗*p <* 0.01, Student’s *t* test. Abbreviations: MASLD, metabolic dysfunction–associated steatotic liver disease; MAVS, mitochondrial antiviral-signaling protein; NAS, nonalcoholic fatty liver disease activity score.

MAVS expression was evaluated in liver biopsies from people with MASLD (nonalcoholic fatty liver disease activity score [NAS] ≥3) or mild-MASLD livers (NAS ≤2) (Supplemental Table S2, http://links.lww.com/HEP/I457). MAVS mRNA expression was significantly higher in the livers of people with MASLD than in those without the disease (Figure 2A). This result agrees with a report that also found increased MAVS gene expression in people with NASH.^15^ However, another study described that protein levels of MAVS were significantly downregulated in people with MASLD.^16^ To assess whether gene expression correlated with protein levels in our cohort samples, we used 2 different antibodies (Figure 2B, Supplemental Figures S1 and S2, http://links.lww.com/HEP/I457 and Supplemental Materials and Methods, http://links.lww.com/HEP/I457). In line with mRNA expression, anti-MAVS Ab-CellSig showed that protein levels of MAVS were also augmented in the liver of people with MASLD (Figure 2B) and positively correlated with NAS score and serum triglycerides, showing no association with BMI (Figure 2C).

We also analyzed MAVS expression in a public data set (GEO accessions GSE135251), which provides bulk RNA sequencing results from samples of 206 patients with MASLD at various fibrosis stages and 10 healthy liver controls.^17^ The analysis indicated that the MAVS mRNA is significantly upregulated in the liver of people with both MASLD and MASH at different stages (MASH F1–F4) (Figures 2D, E). The analysis of this cohort supports our data, indicating that MAVS levels are increased in MASLD.

### MAVS increases lipid content in human hepatocytes

**FIGURE 3:**
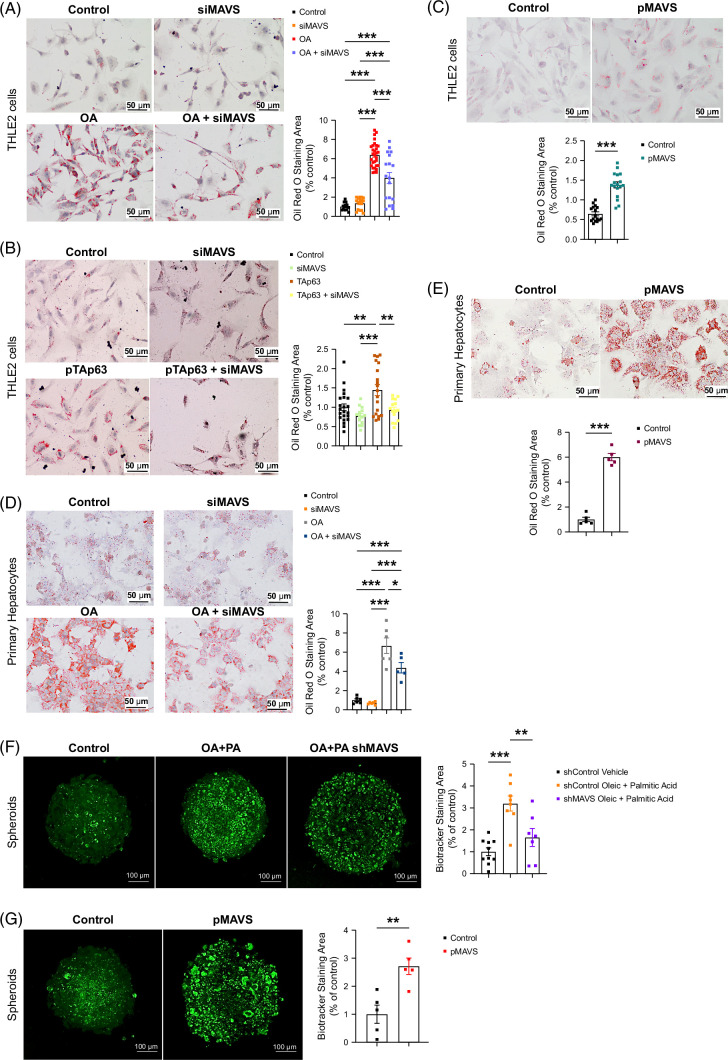
MAVS regulates lipid accumulation in human hepatic cell lines and in mouse primary hepatocytes. (A–C) Representative Oil Red O staining and Oil Red Area of (A) THLE2 cells downregulating MAVS (siMAVS) treated with OA or vehicle for 24 hours (n = 18–30 per group); (B) TAp63 upregulated in THLE2 cells after MAVS silencing (n = 12–23 per group); and (C) THLE2 cells with overexpression of MAVS (n = 16–18 per group). (D, E) Representative Oil Red O staining and Oil Red Area of murine primary hepatocytes with MAVS downregulated treated with OA or vehicle for 24 hours (D) or with MAVS overexpressed (E) (n = 5–6 per group). (F) Spheroids shControl and shMAVS treated with 0.5 mM OA and 0.25 mM palmitic acid. (G) Spheroids with MAVS overexpressed. Data are presented as mean ± SEM; ∗*p* < 0.05, ∗∗*p* < 0.01, ∗∗∗*p* < 0.001, using Student *t* test (C, E, and G) or one-way ANOVA followed by a Bonferroni multiple comparison test (A, B, D, and F). Abbreviations: MAVS, mitochondrial antiviral-signaling protein; OA, oleic acid.

Since both gene expression and protein levels of MAVS were upregulated in the liver of people with MASLD, we studied its impact on hepatocytes. MAVS mRNA expression rose in human hepatic THLE2 cells treated with oleic acid (OA) (Supplemental Figure S3A, http://links.lww.com/HEP/I457). As expected, OA treatment augmented intracellular lipid content; however, siRNA-mediated MAVS silencing reduced OA-induced lipid storage (Figure 3A and Supplemental Figure S3B, http://links.lww.com/HEP/I457). As reported,^4^ TAp63 overexpression increased the lipid droplets in hepatocytes, but this effect was blunted when MAVS was silenced (Figure 3B). Next, we ectopically overexpressed MAVS (Supplemental Figure S3C, http://links.lww.com/HEP/I457) and found an increased lipid content in THLE2 cells (Figure 3C). Parallel treatment with OA and loss-of-function and gain-of-function experiments in primary hepatocytes showed identical results (Figures 3D, E and Supplemental Figures S3D–F, http://links.lww.com/HEP/I457). We also obtained similar results using HepG2 cells: silencing MAVS reduced the OA-induced lipid content while overexpressing MAVS increased the lipid load (Supplemental Figures S3G, H, http://links.lww.com/HEP/I457). Next, a more complex fatty acid mixture was used, combining palmitate acid and OA in THLE2 cells, which caused a significant increase in fatty acid concentration (Supplemental Figure S4A, http://links.lww.com/HEP/I457).

We employed OA and palmitic acid in a 3D AML12 cell spheroid model. MAVS overexpression and silencing were confirmed (Supplemental Figure S4B, http://links.lww.com/HEP/I457). Oleic acid treatment raised fatty acid levels, mitigated by MAVS silencing (Supplemental Figure S4C, http://links.lww.com/HEP/I457). Oleic acid and palmitate treatment increased lipid content, attenuated by MAVS silencing (Figure 3F). MAVS overexpression in spheroids increased lipid content (Figure 3G). Thus, MAVS manipulation in 3D models mirrors findings in 2D models.

### Inhibition of hepatic MAVS ameliorates CDHFD-induced, MCD-induced, and WD-induced MASLD

We next investigated the in vivo relevance of these findings in mice subjected to either (i) a CDHFD (45% kcal from fat) for 16 weeks; (ii) an MCD diet for 4 weeks; or a WD (45% kJ fat, 15% kJ protein, 43% kJ carbohydrates, and 1.25% cholesterol) for 9 weeks (Supplemental Table S1, http://links.lww.com/HEP/I457).

**FIGURE 4:**
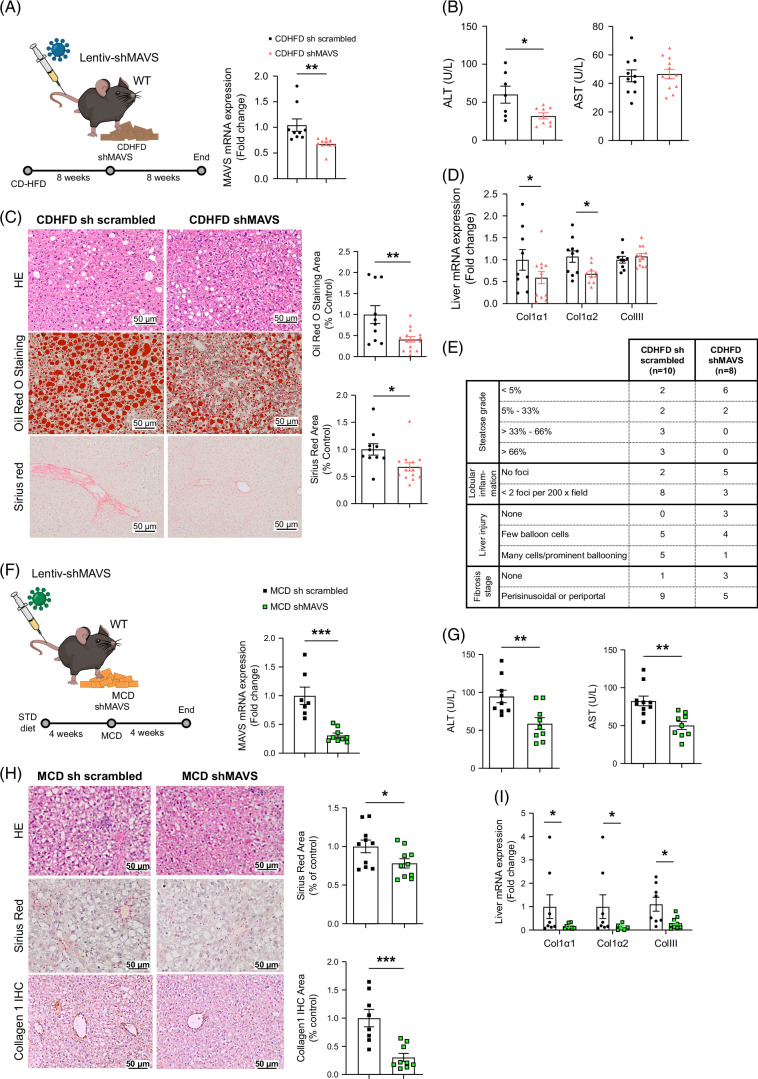
Liver-specific downregulation of MAVS ameliorated lipid accumulation induced by CDHFD or an MCD diet. (A, F) Hepatic mRNA MAVS levels of (A) mice fed a CDFHD for 16 weeks, with TVI of a lentivirus encoding sh-MAVS or shRNA-scrambled control at week 8, as indicated (n = 9–12 per group) and (F) fed an MCD diet for 4 weeks, with TVI of a lentivirus encoding shMAVS or shRNA-scrambled control at first week (n = 7–10 per group). (B, G) Serum levels of ALT and AST, respectively, in mice in the conditions as in (A) and (F). (C) Representative microphotographs of hematoxylin and eosin (upper panel), Oil Red O staining (middle panel), and Sirius Red staining area (lower panel) of liver sections of mice from (A). (H) Representative microphotographs of hematoxylin and eosin (upper panel), Sirius Red staining area (middle panel), and collagen 1 IHC-stained sections (brown area) of liver sections of mice from (F). Oil Red O staining, collagen deposition, and Sirius Red–stained sections were quantified using ImageJ. (D, I) Hepatic mRNA levels of collagen type 1 alpha 1, collagen type 1 alpha 2, and collagen type 3 in mice in the conditions as in (A) and (F). (E) Liver qualitative analyses for steatosis grade, lobular inflammation, liver injury, and fibrosis stage. HPRT was used to normalize mRNA levels. Data are presented as mean ± SEM; *p < 0.05, **p < 0.01, ***p < 0.001 using a Student *t* test. Abbreviations: CDHFD, choline-deficient plus high-fat diet; IHC, immunohistochemistry; MAVS, mitochondrial antiviral-signaling protein; MCD, methionine- and choline-deficient.

Mice received tail vein injections of a lentivirus encoding for a scrambled shRNA or shRNA against MAVS to decrease MAVS expression as described.^18^ Injections were administered either 8 weeks into the CDHFD regime (Figures 4A), 4 weeks before the MCD diet (Figure 4F), or at the first week of WD (Supplemental Figure S6A, http://links.lww.com/HEP/I457). As expected, mice fed a CDHFD showed a very significant weight gain compared to mice fed a chow diet, but no differences were detected when MAVS was knocked down (Supplemental Figure S5A, http://links.lww.com/HEP/I457). Similar results were observed for energy intake (Supplemental Figure S5B, http://links.lww.com/HEP/I457). Serum levels of triglycerides, cholesterol, and non-esterized free fatty acids were also similar between CDHFD scrambled and CDHFD shMAVS groups (Supplemental Figure S5C, http://links.lww.com/HEP/I457). Cleaved caspase 3 and Ki-67 staining showed no significant differences, indicating independent apoptosis or proliferation alterations (Supplemental Figure S5D, http://links.lww.com/HEP/I457). We have also performed a GTT and ITT, and as expected, mice fed a CDHFD are glucose-intolerant and insulin-resistant compared to mice fed a chow diet (Supplemental Figures S5E, F, http://links.lww.com/HEP/I457). MAVS knockdown in the liver of CDHFD-fed mice slightly improved glucose tolerance, and there was a tendency (not significant) to enhance insulin sensitivity.

In mice on CDHFD with MAVS knockdown, circulating ALT was significantly lower (Figure 4B), as compared to mice fed a CDHFD sh scrambled. The liver sections of MAVS knockdown mice also had lower levels of lipids and collagen content (as shown by Oil Red O and Sirius Red staining) (Figure 4C) and lower mRNA expression of fibrosis markers, such as collagen 1 α1 and collagen 1 α2 (Figure 4D). The scoring system of qualitative analyses for steatosis grade, lobular inflammation, liver injury, and fibrosis stage showed that the inhibition of MAVS in the liver improved the liver status (Figure 4E).

We then analyzed whether the MAVS knockdown alleviates MCD diet–induced fibrosis (Figure 4F). MCD diet–fed mice showed the expected reduction of weight gain, but no differences were detected upon MAVS knockdown (Supplemental Figure S5G, http://links.lww.com/HEP/I457). Energy intake (Supplemental Figure S5H, http://links.lww.com/HEP/I457), serum levels of triglycerides, cholesterol, and non-esterized free fatty acids (Supplemental Figure S5I, http://links.lww.com/HEP/I457) and staining for cleaved caspase 3 and Ki-67 (Supplemental Figure S5J, http://links.lww.com/HEP/I457) were also similar between groups. However, there was a reduction in serum AST and ALT in the knockdown mice as compared to the control (shRNA-scrambled) mice (Figure 4G). In agreement with this, liver from MAVS knockdown mice showed lower levels of collagen content, collagen 1 staining (Figure 4H), and expression of profibrotic markers (Figure 4I).

We also inhibited MAVS in the liver of WD-fed mice (Supplemental Figure S6A, http://links.lww.com/HEP/I457). As predicted, WD-fed mice gained more body weight compared to mice fed a chow diet, but no differences were detected upon hepatic MAVS knockdown (Supplemental Figure S6B, http://links.lww.com/HEP/I457). WD-fed mice also displayed increased weight and feeding, while MAVS inhibition had no effect (Supplemental Figure S6B, http://links.lww.com/HEP/I457). WD-fed mice also displayed a significant glucose intolerance (Supplemental Figure S6C, http://links.lww.com/HEP/I457) and insulin resistance (Supplemental Figure S6D, http://links.lww.com/HEP/I457), which were improved upon hepatic knockdown of MAVS (Supplemental Figures S6C, D, http://links.lww.com/HEP/I457), as well as reduced circulating AST and ALT, collagen content (as shown by Sirius Red staining), and the expression of markers of fibrosis and inflammation when compared to control mice fed a WD (Supplemental Figures S6E–I, http://links.lww.com/HEP/I457).

### Inhibition of hepatic MAVS ameliorates TAp63-induced steatosis

We analyzed whether MAVS inhibition would affect TAp63-induced steatosis in mice. Mice on a standard diet received tail vein injections with AAV8-TAp63 or AAV8-GFP. After 8 weeks, they were injected again with either shMAVS or shRNA-scrambled (as a control) and maintained on the same diet for another 8 weeks. The administration of AAV8-TAp63 or lentivirus-shMAVS led to increased p63 or decreased MAVS levels, respectively (Supplemental Figure S7A, http://links.lww.com/HEP/I457). Body weight, energy intake (Supplemental Figure S7B, http://links.lww.com/HEP/I457), glucose tolerance, and insulin sensitivity remained unchanged (Supplemental Figures S7C, D, http://links.lww.com/HEP/I457). While liver mass, serum triglycerides, and cholesterol were similar among all groups, non-esterized free fatty acids were reduced when MAVS levels were reduced (Supplemental Figure S7E, http://links.lww.com/HEP/I457). As reported, the overexpression of TAp63 caused liver steatosis,^4^ an effect blunted by MAVS knockdown (Supplemental Figure S7F, http://links.lww.com/HEP/I457).

### The specific inhibition of MAVS in hepatocytes protects against MCD-induced and WD-induced MASLD

We next tested whether the specific ablation of MAVS in only hepatocytes would be sufficient to ameliorate MASLD in mice fed an MCD diet for 4 weeks or mice fed a WD for 9 weeks (Supplemental Table S1, http://links.lww.com/HEP/I457).

**FIGURE 5:**
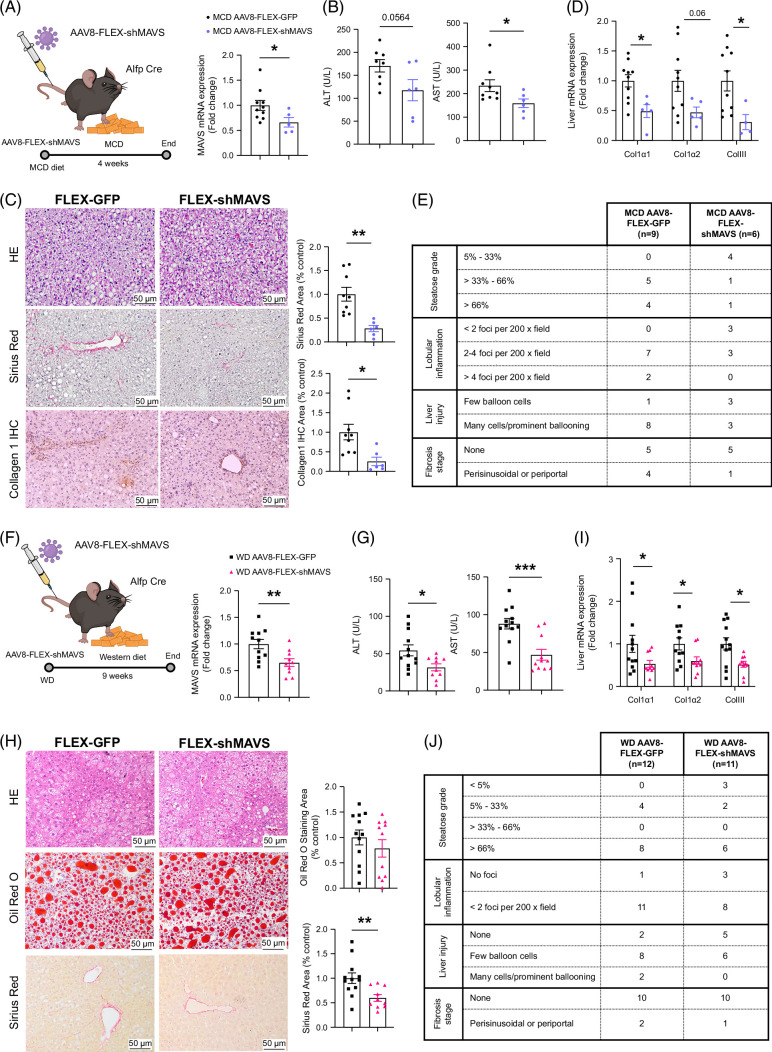
Hepatocyte-specific downregulation of MAVS ameliorates steatohepatitis induced by methionine-and-choline-deficient (MCD) diet. (A, F) Hepatic mRNA MAVS levels of mice fed an MCD diet for 4 weeks, which received tail vein injection of AAV8-FLEX-shMAVS or AAV8-FLEX-GFP as indicated (n = 6–9 per group) and of mice fed a Western diet (WD) for 9 weeks, which received tail vein injection of AAV8-FLEX-shMAVS or AAV8-FLEX-GFP as indicated (n = 11–12 per group), respectively. (B, G) Serum levels of ALT and AST of mice AAV8-FLEX-shMAVS or AAV8-FLEX-GFP from (A) and (F). (C) Representative microphotographs of hematoxylin and eosin (upper panel), Sirius Red area (middle panel), and collagen type 1 IHC (lower panel) of liver sections of mice from (A). (H) Representative microphotographs of hematoxylin and eosin (upper panel), Oil Red O staining area (middle panel), and Sirius Red–stained sections (pink area) of liver sections of mice from (F). Oil Red O staining, collagen deposition, and Sirius Red–stained sections were quantified using ImageJ. Collagen deposition and Sirius Red–stained sections (pink area) (bottom panel) were quantified using ImageJ (H). (D, I) Hepatic mRNA levels of collagen type 1 alpha 1, collagen type 1 alpha 2, and collagen type 3. (E, J) Liver qualitative analyses for steatosis grade, lobular inflammation, liver injury, and fibrosis stage. HPRT was used to normalize mRNA levels. Data are presented as mean ± SEM; *p < 0.05, **p < 0.01, ***p < 0.001, Student *t* test. Abbreviation: MAVS, mitochondrial antiviral-signaling protein.

In the first model, MAVS mRNA expression was effectively downregulated in mice after AAV8-FLEX-shMAVS treatment as compared to the control-treated mice (with AAV8-FLEX-GFP) fed with MCD diet for 4 weeks (Figure 5A). As expected, once the mice were fed the MCD diet, they gradually lost weight. This is due to a vastly lower caloric intake and alterations in fatty acid metabolism,^19–21^ but the inhibition of hepatic MAVS did not alter body weight or feeding (Supplemental Figure S8A, B, http://links.lww.com/HEP/I457). Strikingly, the hepatocyte-specific MAVS knockdown led to a similar phenotype of the mouse liver as when MAVS was inhibited in all liver cell types: circulating ALT and AST, the Sirius Red area, collagen 1 staining, and gene expression of fibrosis markers were reduced as compared to those in the control mice (Figures 5B–D). Also, steatosis grade, lobular inflammation, liver injury, and fibrosis stage were overall improved (Figure 5E). Of note, these effects were not related to cellular proliferation or apoptosis, as the immunostaining of Ki-67 and CC3 was similar in both the MAVS knockdown and control knockdown mice fed an MCD diet (Supplemental Figure S8C, http://links.lww.com/HEP/I457).

In the second model, we specifically inhibited MAVS in hepatocytes of mice fed a WD for 9 weeks. MAVS mRNA expression was significantly downregulated in mice after AAV8-FLEX-shMAVS treatment as compared to the control-treated mice (with AAV8-FLEX-GFP) (Figure 5F). Weight gain and energy intake were unaffected after the inhibition of MAVS in the liver (Supplemental Figures S8E, F, http://links.lww.com/HEP/I457). The knockdown of hepatic MAVS improved glucose tolerance (Supplemental Figure S8G, http://links.lww.com/HEP/I457) and insulin sensitivity (Supplemental Figure S8H, http://links.lww.com/HEP/I457) in comparison to mice fed a WD. The inhibition of MAVS in the liver caused a significant downregulation of circulating AST and ALT (Figure 5G), the collagen content (as shown by Sirius Red staining) (Figure 5H), and the expression of markers of fibrosis (Figure 5I) when compared to control mice fed a WD. Moreover, steatosis grade, lobular inflammation, liver injury, and fibrosis stage were overall improved (Figure 5J). Again, these effects were independent of cellular proliferation or apoptosis (Supplemental Figure S8I, http://links.lww.com/HEP/I457).

### MAVS increases hepatocyte lipid content through the ERK/TNFα/NFκβ signaling pathway

**FIGURE 6:**
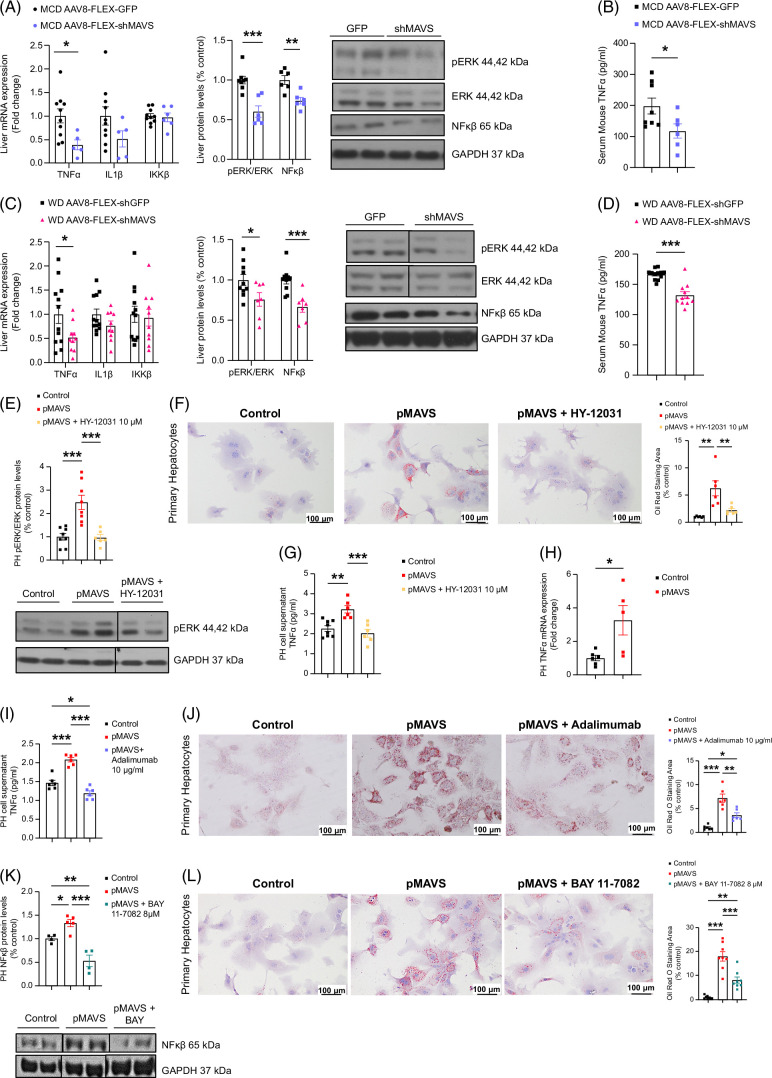
The effects of MAVS on lipid metabolism were modulated by proinflammatory ERK/TNFα/NF-κβ pathway. (A, C) Hepatic mRNA levels of TNFα, IL1β and IKKβ, hepatic protein levels of pERK/ERK and NFκβ, protein levels of and (B, D) serum concentration of TNFα of mice fed an MCD diet for 4 weeks, which received tail vein injection of AAV8-FLEX-shMAVS or AAV8-FLEX-GFP as indicated (n = 5–9 per group) and of mice fed a Western diet (WD) for 9 weeks, which received tail vein injection of AAV8-FLEX-shMAVS or AAV8-FLEX-GFP as indicated (n = 7–11 per group. (E, F, G) pERK/ERK protein levels, representative microphotographs of Oil Red O staining and Oil Red area, and TNFα cell supernatant concentration of primary hepatocytes cells with overexpression of MAVS with or without HY-12031 10 µM 24 hours (ERK inhibitor) (n = 6–8 per group). (H, I, J) mRNA levels of TNFα, TNFα cell supernatant concentration, and representative microphotographs of Oil Red O staining and Oil Red area of primary hepatocytes cells with overexpression of MAVS with or without adalimumab 10 µg/mL for 24 hours (TNFα inhibitor) (n = 6 per group). (K, L) NFκβ protein levels and representative microphotographs of Oil Red O staining and Oil Red area of primary hepatocytes with overexpression of MAVS with or without BAY 11-7082 8 µM for 24 hours (NFκβ inhibitor) (n = 5–8 per group). HPRT was used to normalize mRNA levels, and GAPDH was used to normalize protein levels. Data are presented as mean ± SEM; *p < 0.05, **p < 0.01, ***p < 0.001, Student *t* test (A–D) and one-way ANOVA followed by a Bonferroni multiple comparison test (E–K). Abbreviation: MAVS, mitochondrial antiviral-signaling protein.

The MAVS signaling pathway stimulates the expression of proinflammatory cytokines,^22,23^ which are implicated in the development of MASLD.^24^ We evaluated inflammatory markers by measuring hepatic TNFα, IL-1β, IKKβ, and NFκβ expression in control or MAVS knockdown mice fed an MCD diet. MAVS inhibition reduced TNFα and IL-1β mRNA and NFκβ protein levels in the liver (Supplemental Figures S5K, L, http://links.lww.com/HEP/I457), along with decreased TNFα levels (Supplemental Figure S5M, http://links.lww.com/HEP/I457), compared to control MCD-fed mice. We performed identical measurements in mice fed an MCD diet but with hepatocyte-specific MAVS deletion. Similarly, these mice displayed decreased hepatic TNFα and NFκβ expression and circulating TNFα levels (Figures 6A, B).

It is widely documented that inhibiting p38 MAPK and Erk decreases TNFα synthesis.^25–28^ Also, JNK is required for TNFα expression.^29^ To investigate whether MAVS modulates these kinases, influencing TNFα levels and hepatic inflammation, we measured pJNK/JNK, pERK/ERK, and pp38 protein levels in our mouse models with manipulated hepatic MAVS. In mice fed an MCD diet with hepatic MAVS knockdown, pJNK/JNK and pp38 levels remained unchanged, but the pERK/ERK ratio decreased (Supplemental Figures S5L, N, http://links.lww.com/HEP/I457). Similar results were observed when MAVS was inhibited in hepatocytes of MCD-fed mice (Figure 6A and Supplemental Figure S8D).

Then, we measured hepatic TNFα, IL-β, and IKKβ expression in control mice and those with hepatic MAVS knockdown, fed either a control or WD. We observed reduced TNFα and IL-β1 mRNA in the liver, along with reduced circulating TNFα levels (Supplemental Figure S6H, http://links.lww.com/HEP/I457) compared to sh-scrambled injected mice fed a WD. Consistently, pERK/ERK and NFκβ protein levels significantly declined following hepatic MAVS knockdown (Supplemental Figure S6I, http://links.lww.com/HEP/I457).

Hepatic expression of TNFα, IL-β1, IKKβ, and NFκβ was measured in the livers of WD-fed mice with hepatocyte-specific MAVS inhibition. Like previous results, hepatic and circulating TNFα levels were decreased upon MAVS silencing (Figures 6C, D). The pERK/ERK ratio was also significantly lower in the liver of mice with inhibited MAVS (Figure 6C). These results indicate that MAVS induces ERK phosphorylation in the liver and thereby increases TNFα expression.

We next assessed the regulation of the ERK-TNFα signaling pathway in in vitro models. To ascertain whether ERK phosphorylation regulates the lipid buildup induced by MAVS, we treated hepatocytes with an ERK inhibitor, namely U0126/HY-12031. MAVS overexpression significantly increased pERK/ERK, and the treatment with HY-12031 completely abolished ERK phosphorylation (Figure 6E). Upon lipid content assessment, MAVS caused the expected rise in lipid droplet quantity, and the ERK inhibitor normalized lipid content to basal levels (Figure 6F). In addition, secreted TNFα levels in the supernatant were heightened following MAVS overexpression and attenuated when cells were treated with HY-12031 (Figure 6G).

To address whether the increase of TNFα mediates the MAVS-induced lipid accumulation, we treated primary hepatocytes with adalimumab, a TNFα monoclonal antibody approved for the treatment of rheumatoid arthritis.^30^ We found that TNFα mRNA levels in primary hepatocytes with MAVS overexpression were significantly upregulated (Figure 6H), and indeed, adalimumab blunted MAVS-induced TNFα supernatant levels (Figure 6I). These results were concomitant with the blockade of MAVS-induced lipid storage (Figure 6J). We also overexpressed MAVS in primary hepatocytes and treated them with the inhibitor of κB kinase, BAY 11-7082.^31^ This pharmacological agent also inhibited MAVS-induced NFκβ (Figure 6K) and fatty acid content in hepatocytes (Figure 6L). Overall, these results indicate that the ERK/TNFα/NFκβ pathway regulates the deleterious effects of MAVS in hepatocytes.

### MAVS regulates the metabolism of fatty acids and bile acids

Once hepatic MAVS levels’ significance in modulating diet-induced liver inflammation was established, we aimed to evaluate markers of de novo lipogenesis, fatty acid oxidation, and fatty acid uptake to ascertain if fatty acid metabolism could mediate MAVS-induced lipid load. We assessed gene expression involved in fatty acid metabolism in mice fed an MCD diet with whole-liver MAVS deletion. However, no differences were observed in any studied genes (data not shown). Fatty acid oxidation was also measured in THLE2 cells with MAVS overexpression or silencing, yet no significant differences were found (data not shown). Subsequently, a proteomic assay was conducted in primary hepatocytes with MAVS overexpression, revealing 21 downregulated and 15 upregulated proteins (Supplemental Figures S9A, B, http://links.lww.com/HEP/I457). Notably, proteins associated with reverse cholesterol transport and bile acid (BA) secretion were enriched (Supplemental Figure S9C, http://links.lww.com/HEP/I457).

Disruption of BA homeostasis is closely linked to the progression of MASLD, contributing to dysregulated energy balance, increased liver inflammation, and fibrosis. Elevated levels of BA, observed in individuals with NASH, suggest a correlation between toxic BA levels and MASH development.^32,33^ Therefore, our proteomic data may suggest that the MAVS-induced liver steatosis is somehow mediated by BAs. Indeed, future studies are needed to elucidate the precise interaction between MAVS and BAs, along with the functional relevance of BA as mediators of the deleterious effects of MAVS in the liver.

### *O*-GlcNAcylation of MAVS induces hepatocyte lipid content and inflammation

**FIGURE 7:**
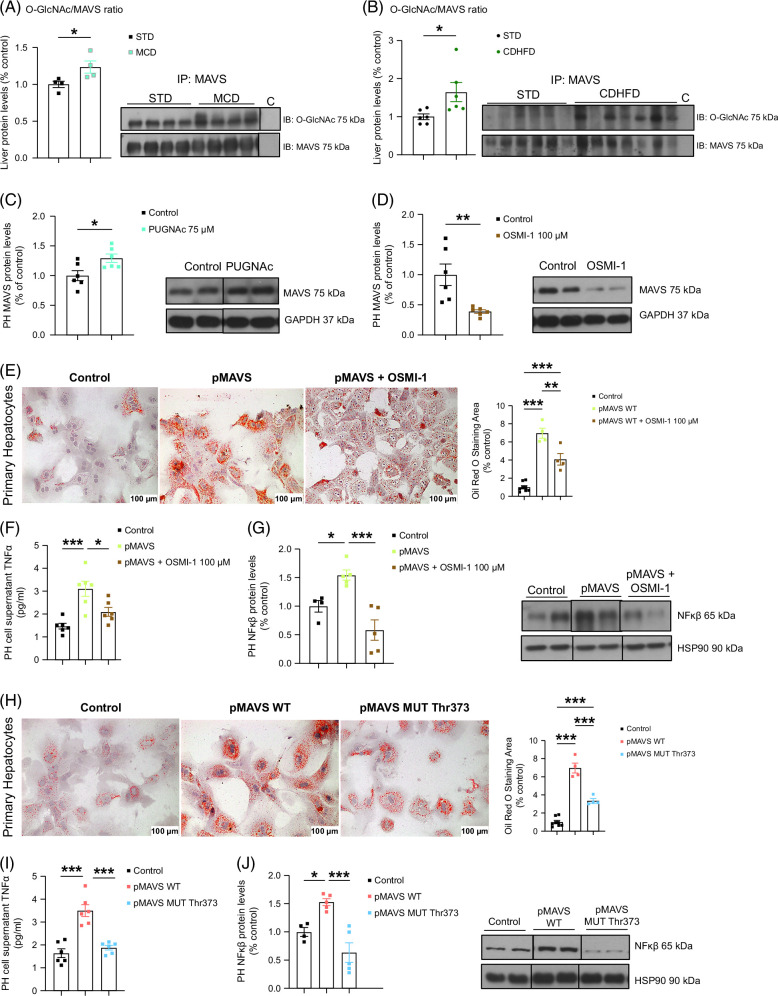
*O*-GlcNAcylation of MAVS regulates lipid accumulation. (A) *O*-GlcNAcylated levels of MAVS in the liver of mice fed STD and MCD diet (n = 4 per group). (B) *O*-GlcNAcylated levels of MAVS in the liver of mice control and CDHFD (n = 6 per group). (C, D) MAVS protein levels of primary hepatocyte cells in the absence or presence of PUGNAc 75 µM and OSMI-1 100 µM for 24 hours (n = 6 per group). (E, F, G) Representative Oil Red O staining, TNFα release to cell supernatant, and NFκβ protein levels of primary hepatocytes cells with MAVS overexpression treated with OSMI-1 100 µM or vehicle for 24 hours (n = 4–6 per group). (H, I, J) Representative Oil Red O staining, TNFα release to cell supernatant, and NFκβ protein levels of primary hepatocyte cells with MAVS overexpression and MAVS Mutant Thr373 overexpression (n = 4–8 per group). GAPDH was used to normalize protein levels. Data are presented as mean ± SEM; *p < 0.05, **p < 0.01, ***p < 0.001, Student *t* test (A–D) and one-way ANOVA followed by a Bonferroni multiple comparison test (E–J). Abbreviations: CDHFD, choline-deficient plus high-fat diet; MAVS, mitochondrial antiviral-signaling protein; MCD, methionine- and choline-deficient.

*O*-GlcNAcylation of MAVS is crucial in mediating its different actions,^11,14^ and we have recently linked *O*-GlcNAcylation to MASLD development.^34^ We investigated whether this posttranslational modification modulates MAVS effects on inflammation and steatosis. Coimmunoprecipitation analysis showed increased *O*-GlcNAcylated MAVS in the livers of MCD diet–fed mice (Figure 7A) and CDHFD-fed mice (Figure 7B). Thus, we treated primary hepatocytes with *O*-(2-acetamido-2-deoxy-d-glucopyransylidene)-amino-*N*-phenylcarbamate (PUGNAc), an inhibitor of *O*-GlcNAcase, the enzyme that prevents the removal of *O*-GlcNAc form target proteins and therefore increases protein *O*-GlcNAc levels,^35^ and found that PUGNAc increased MAVS protein levels (Figure 7C). We then treated primary hepatocytes with OSMI-1, an inhibitor of *O*-GlcNAc transferase that catalyzes the addition of *O*-GlcNAc to target proteins and thus decreases protein *O*-GlcNAc levels,^36^ which reduced MAVS protein levels (Figure 7D). We ectopically overexpressed MAVS and, after 24 hours, treated them with OSMI-1. As in experiments described above, MAVS favored fatty acid accumulation; however, the treatment with OSMI-1 blunted MAVS-induced hepatocyte lipid content (Figure 7E), and this was associated with lower levels of TNFα (Figure 7F) and NFκβ (Figure 7G).

Finally, to gain insight into the functional consequences of *O*-GlcNAcylation of MAVS, we identified MAVS *O*-GlcNAcylation sites using the software https://www.oglcnac.mcw.edu/. Thr373 was the site identified in mice and therefore, we mutated Thr373 to Ala. Wild-type MAVS increased lipid content and secreted TNFα and NFκβ protein levels in primary hepatocytes. In contrast, the T373A-MAVS mutant completely lost its capacity to exert those effects (Figures 7H–J). Overall, these data demonstrate that *O*-GlcNAcylation in the Thr373 is required for the action of MAVS on hepatic inflammation and fatty acid content.

## DISCUSSION

In this work, we have identified the role of MAVS on fatty acid metabolism and its implications in the development of MASLD. MAVS is elevated in the liver of animal models as well as of people with MASLD. Moreover, our in vitro and in vivo genetic functional studies indicated that overexpression of MAVS induces lipid deposition while its silencing alleviates steatosis.

To our knowledge, the regulation of MAVS in human MASLD has been previously assessed in only 2 studies. The first study showed that MAVS gene expression is increased in the liver of people with NASH as compared with a non-NASH control group^15^; however, the second study found that the MAVS protein levels are reduced in the liver of people living with MASLD.^16^ In our study, we found that both mRNA and protein levels of MAVS are significantly upregulated in people with MASLD, with MAVS transcripts being positively associated with NAS and serum triglyceride levels. Two critical points could explain these apparently discrepant results. First, MASLD characterization and sample size of the cohorts differed: we used 18 samples from people with obesity and MASLD (in fibrosis stage 1 or 2) diagnosed according to Brunt-Kleiner’s criteria^37^; in contrast, in the previous report, 8 samples with an NAS ≥3 are classified as MASLD, while those with NAS ≤2 without steatosis are classified as non-MASLD,^18^ and the potential impact of dyslipidemia on MAVS expression was not analyzed. Second, 2 different antibodies were used: while both antibodies showed a clear reduction in protein levels after silencing MAVS in a human hepatic cell line, the antibody used in the previous study did not show differences when MAVS was overexpressed, and when tested in human samples questions about its reliability emerge (Supplemental Figure S1, http://links.lww.com/HEP/I457). Finally, different protocols for protein extraction were likely used, which may affect the quality of the western blots. Further studies will be necessary to clarify these issues. Indeed, the observed discrepancies may stem from the utilization of small cohorts in both studies. To substantiate our findings across a broader spectrum, we leveraged a publicly accessible data set from what we believe to be one of the largest cohorts available, which offers comprehensive transcriptomic data.^17^ The analysis indicated that the mRNA expression of MAVS is significantly upregulated in the liver of people with both MASLD and MASH at different stages (MASH F1–F4), supporting that MAVS levels are increased in MASLD.

In addition to its regulation, the other key question is whether the inactivation of MAVS is beneficial or detrimental to the liver. To address this question, a study using mouse models with either MAVS global knockout or hepatocyte-specific MAVS knockout reported that these mice were more prone to developing diet-induced NAFLD.^16^ In contrast, we found in this study that adult-onset MAVS inhibition in the whole liver or specifically in hepatocytes protected mice against diet-induced MASLD. However, there is a fundamental difference between the 2 studies: while the earlier work disrupted MAVS from embryonic stages, we knocked down MAVS in adult stages after liver damage had already been induced. Therefore, the deletion of MAVS seems to exert very different actions in mice depending on the time of the genetic intervention and liver status: its deletion is detrimental at early stages in healthy conditions, but it is beneficial when the mice have already developed MASLD.

MAVS is widely recognized as playing an essential role in antiviral innate immunity, whereby it mediates the activation of NFκB and interferons and the induction of interferons in response to viral infection in multiple cell types, prompting the secretion of proinflammatory cytokines.^9,38^ For instance, the deletion of MAVS abolishes the induction of IFN-I and other proinflammatory cytokines by respiratory syncytial virus.^39^ Inflammation is also one of the hallmarks of MASH and is considered paramount for disease progression.^40,41^ Under conditions of stress, the expression of inflammatory cytokines, such as TNFα, is increased in hepatocytes.^42^ The current dogma is that both hepatocytes and Kupffer cells are the primary sources of TNFα production in the initiating stage of MASH, whereas infiltrated monocytes and macrophages later contribute to the vicious cycle of TNFα-signaling cascade in MASH progression.^43^ Our results indicate that MAVS induces the expression and release of TNFα in hepatocytes and its downregulated transcription factor NFκβ, and that this inflammatory signaling pathway is an important mediator of the adverse effects caused by MAVS, as its inhibition blunted MAVS-induced steatosis. Since TNFα is known to be modulated by kinases such as p38 MAPK, ERK, and JNK,^25–29^ we tested whether these factors could regulate the actions of MAVS on TNFα activation. The data indicate that ERK is a key player since its inhibition blunted MAVS-induced TNFα elevation. Although our study focused on hepatocytes, our results do not discard that in vivo, at least part of the effects could also be mediated by TNFα produced in other cells (eg, Kupffer cell, macrophages, or monocytes).

Many studies have shown that MAVS can be regulated by posttranscriptional and posttranslational modifications, which affect its function in promoting innate immune responses (reviewed in Ren et al^44^). One posttranslational modification is glycosylation by *O*-linked *N*-acetylglucosamine (*O*-GlcNAc) transferase (OGT), which activates MAVS and is crucial for its antiviral response.^11^ Our lab has recently demonstrated that *O*-GlcNAcylation is also an important feature in the development of MASLD, as OGT inhibits mitochondrial function, favoring the storage of fatty acids in hepatocytes, while the genetic knockdown of OGT in animal models of MASLD alleviates the disease.^34^ In the present study, we found that MAVS is hyper-*O*-GlcNAcylated in the liver of mice with MASLD and that the pharmacological inhibition of OGT blunted MAVS-induced lipid storage. This is because the suppression of *O*-GlcNAcylation ameliorated MAVS-induced NFκβ expression and TNFα secretion in hepatocytes. Importantly, when MAVS was mutated in Thr373, the actions of MAVS on inflammation and lipid accumulation did not occur. Therefore, our results indicate that this posttranslational modification regulates the harmful action of MAVS in hepatocytes.

In summary, our findings show that: (a) MAVS expression is increased in the liver of mouse models of MASLD and of people living with MASLD; (b) MAVS overexpression induced lipid accumulation; (c) inhibition of MAVS in the whole liver or specifically in hepatocytes of adult mice ameliorated TAp63α-induced and diet-induced liver steatosis; (d) the steatotic action of MAVS is mediated by ERK/TNFα/NFκβ; and (e) the posttranslational modification *O*-GlNAcylation is critical for MAVS-induced inflammation and lipid storage. Notably, we present preliminary functional data demonstrating that silencing MAVS genetically or inhibiting its *O*-GlcNAcylation through pharmacological means effectively prevents steatosis and MASLD development. Overall, our results point toward MAVS as a molecule implicated in the development of steatosis.

## Supplementary Material
