# Supplementary material for: Mitochondrial antiviral signaling protein enhances MASLD progression through the ERK/TNFα/NFκβ pathway
Source: Hepatology. 2024 May 16;81(5):1535–52. doi: 10.1097/HEP.0000000000000930 (PMC11999095; doi:10.1097/HEP.0000000000000930)
Supplement: Supplementary file 1 [file hep-81-1535-s001.pdf]

Supplemental Materials and Methods

Animals and diets.

Mice experiments were conducted according to the standard protocols approved by the Animal Ethics Committee at the University of Santiago de Compostela, and mice received the highest levels of humane care. C57BL/6Jmice were housed in rooms under constant temperature (22 °C), with a 12:12 h light/dark cycle. When the mice were 8 weeks old, they were given *ad libitum* access to a standard diet (SD) (6% kcal fat, 18% kcal protein, Teklad Global, Inotiv), a choline-deficient high-fat diet (CDHFD) (D05010402; 45 kcal% fat, Research Diets), a methionine- and choline-deficient (MCD) diet (A02082002BR, Research Diets), a high-fat diet (HFD) (D12492; 60% kcal fat, Research Diets) or Western diet (TD88137; 42 % kJ fat, 15% kJ protein, 43% kJ carbohydrates and 1.25% cholesterol, Ssniff and its recommended chow diet (CD) (CD88137; 13% kJ fat, 18% kJ protein, 69% kJ carbohydrates, Ssniff) used during specified times and experiments. The macronutrients composition of each diet is indicated in Supplementary Table 1. Food intake and body weight were measured weekly during all experimental phases. After the animals were euthanized, serum and liver samples were collected. All molecular and histological studies were conducted using fragments from the left lateral lobe.

Cohort of patients with NASH for western blot analysis.

Liver biopsies were obtained intra-operatively from patients with severe obesity undergoing bariatric surgery (n=18) at the Clínica Universidad de Navarra (Spain). Obesity was defined as a BMI≥30 kg/m2 and body fat percentage as ≥35%. BMI was calculated as weight in kilograms divided by the square of height in meters, and body fat

was estimated by air-displacement plethysmography (Bod-Pod®, Life Measurements, Concord, CA, USA). Inclusion criteria encompassed a complete diagnostic work-up including physical examination, laboratory investigation, ultrasound echography, and liver biopsy, consistent with the diagnosis of NASH according to the criteria of Kleiner and Brunt by an expert pathologist masked to all results of the assays (1). Features of steatosis, lobular inflammation, and hepatocyte ballooning were combined to obtain a MASLD activity score (NAS) (0-8). Exclusion criteria were: a) daily alcohol intake ≥20 g for women and ≥30 g for men; b) evidence of hepatitis B virus surface antigen or hepatitis C virus antibodies in the absence of a history of vaccination; c) use of drugs causing MASLD (i.e. amiodarone, valproate, tamoxifen, methotrexate, corticosteroids or anti-retrovirals); and d) presence of other specific liver diseases, such as autoimmune liver disease, haemochromatosis, Wilson's disease, or α-1-antitrypsin deficiency. Anthropometric, biochemical and clinical characteristics is described in Supplementary Table 2. All reported investigations were carried out in accordance with the principles of the Declaration of Helsinki, as revised in 2013, and approved by the Hospital's Ethical Committee responsible for research (protocol 2021.005). Written informed consent was obtained from all the participants.

Quantitative Proteomic by SWATH method.

Protein extraction and digestion. Proteins from liver samples or mouse primary hepatocytes were prepared by homogenization using a TissueLyser II (Qiagen) in cold RIPA buffer [containing 200 mmol/l Tris/HCl (pH 7.4), 130 mmol/l NaCl, 10%(v/v) glycerol, 0.1%(v/v) SDS, 1%(v/v) Triton X-100, 10 mmol/l $MgCl_2$] with anti-proteases and anti-phosphatases (Sigma-Aldrich). The tissue lysates were centrifuged for 20 min at 14,000 g in a microfuge at 4 °C. The protein quantification was assessed for each sample

using RC- DC kit (Bio-Rad) according to manufacturer's recommendations. 100 μg from each sample from each model was loaded on a 10% SDS-PAGE gel to whole-protein in gel concentration. Gel was staining and the band was exscinded and submitted to an in gel tryptic digestion. Peptides were extracted by carrying out three 20-min incubations in 40 μl of 60% acetonitrile dissolved in 0.5% HCOOH, then pooled, concentrated (SpeedVac), and stored at −20 °C. Mass spectrometric analysis by SWATH (Sequential Window Acquisition of all Theoretical Mass Spectra). In order to construct the MS/MS spectral libraries each sample was equally pooled in control group and TAp63 overexpressed group (n=3 per group) or control group and p63 downregulated group (n=3 per group) and empty plasmid (n=6) or pMAVS (n=6). All peptide solutions were analyzed by a shotgun data-dependent acquisition (DDA) approach by micro-LC-MS/MS, an analysis as previously described (2, 3). Therefore, ion density found in the DDA runs was used to create the necessaries windows in the SWATH method. Once the SWATH method is created the individual samples were run as described previously by our group (2, 3). The targeted data extraction from the SWATH runs was performed by PeakView (version 2.2) using the SWATH Acquisition MicroApp (version 2.0) and the data was processed using the spectral library created previously from DDA runs. SWATH quantization was attempted for all proteins in the ion library that were identified by ProteinPilot with an FDR below 1%. PeakView computed an FDR and a score for each assigned peptide according to the chromatographic and spectra components; only peptides with an FDR below 1% 10 peptides and 7 transitions per peptide were used for protein quantization. The integrated peak areas were processed by MarkerView software (AB SCIEX) for data-independent method for relative quantitative analysis. To control for possible uneven sample loss across the different samples during the sample preparation process, we performed a MLR normalization (Most Likely Ratio).

Unsupervised multivariate statistical analysis using principal component analysis (PCA) was performed to compare the data across the samples. A Student's t-test analysis on the averaged area sums of all the transitions derived for each protein in every sample will indicate how well each variable distinguishes the two groups, reported as a p-value. For each library, its set of differentially expressed proteins (p-value <0.05) with a 1.2-fold in- or decrease was selected. Functional analysis was performed using the online Reactome software and FunRich3.1.3 software (Functional Enrichment Analysis Tool). Volcano plots were performed using GraphPad Prism 8.0.2.

Generation of lentiviral particles.

The specific shRNA sequences for knockdown of shMAVS, or scramble shRNA (targeting Luciferase) were designed using the GPP Web Portal Tool (available at https://portals.broadinstitute.org/gpp/public/). The AAV8-FLEX-shMAVS was used in Alfp-Cre mice experiment. The oligos targeting the transcripts of interest were synthesized and subcloned into pLKO.1 puro GFP vectors (Addgene), following a previously described protocol (4). Briefly, HEK293T cells were cultured in high- glucose DMEM supplemented with 10% FBS (10270106, Gibco), 2 mM L-glutamine, and 1% penicillin and streptomycin. For transfection, cells were plated at a density of 8×10^6 cells per 150 mm dish and transfected 24 h later with PEI (Polyethylenimine; Sigma-Aldrich, 408727), along with 20 µg of pLKO.shRNAs plasmids with 10 µg of psPAX2 and pMD2.G packaging mix. 24 hours later the medium was changed, and virus-containing supernatants were collected 48h and 72h post-transfection. Lentiviral particles were concentrated using centrifugal filter units with a 0.22µm pore size (Amicon, UFC903024). The target sequences of the shRNAs used in this study were:

shMAVS #1: 5'- GCAACCAGACTGGACCAAATA -3',

shMAVS #2: 5'- GCTGAGTCAGAGAAACTTAAA -3',

shLuciferase: 5'-CCTAAGGTTAAGTCGCCCTCG-3'

Tail vein injections for in vivo lentiviral and adenoviral gene delivery.

Mice were held in a specific restrainer for intravenous injections, Tailveiner (TV-150, Bioseb). The injections into the tail veins were carried out using a 27 G x 3/8" (0.40 mm x 10 mm) syringe. Mice were administered with 100 µl of lentiviral MAVS shRNA ($1x10^9$ TU /mL) or control shRNA ($1x10^9$ TU /mL), 100 µl of AAV8-TAp63α ($1x10^9$ TU /mL) or AAV8-GFP ($1x10^9$ TU /mL) and 100 µl of AAV8-loxP shMAVS ($1x10^9$ TU /mL) or AAV8- GFP ($1x10^9$ TU /mL), diluted in saline. In the study using mice fed at CDHFD diet for 16 weeks, the lentivirus shMAVS was injected 8 weeks before the sacrifice. In the study using mice fed a standard diet for 16 weeks, the AAV8-TAp63α vector was injected in the 1st week and the lentivirus shMAVS was injected in the 8th week before feeding them a standard diet for another 8 weeks. In the study using mice fed at MCD diet for 4 weeks, the lentivirus shMAVS and the AAV8-loxP-shMAVS was injected during the 1st week. Animals were killed by decapitation. Tissues were immediately frozen in dry ice and kept at -80 °C until their analysis.

Histological procedures.

Oil Red O staining. Frozen liver samples were cut in 8µm sections with a cryostat and stained in filtered Oil Red O for 10 min. After being washed in distilled water, sections

were counterstained with Mayer´s hematoxylin for 3 min and mounted in aqueous mounting (glycerin jelly).

Hematoxylin and Eosin and staining. Liver samples were fixed in 4% formaldehyde for 24 h and then dehydrated and embedded in paraffin. Sections of 4µm were cut with a microtome and stained using a standard Hematoxylin and Eosin alcoholic procedure according to the manufacturer´s instructions (BioOptica). Then, sections were rinsed with distilled water, dried at 37°C for 30 min and mounted with permanent (non-alcohol, non-xylene based) mounting media.

Sirius Red staining. Samples fixated in paraffin were dewaxed, hydrated and stained in PicroSirius staining red for 1h. Then, samples were washed with distilled water, dehydrated in three changes of 100% ethanol and cleared in xylene and mounted in a resinous medium.

In these three histological staining techniques, up to 4 representative microphotographs of each animal were taken with a BX51 microscope equipped with a DP70 digital camera (Olympus). Lipids in Oil Red O- stained sections, collagen depositions in Sirius Red-stained sections and positive area in immunohistochemistry were quantified using Image J 1.52p software. A similar procedure of Oil Red O staining was performed for THLE2, HepG2 and primary hepatocytes, normalizing the data to the number of nuclei per image.

Immunofluorescence. For MAVS and albumin immunofluorescence staining, samples fixated in paraffin were dewaxed, hydrated, pre-treated with PT link DAKO 20min 97º in Tris EDATA buffer at pH 9, washed with PBS and incubated for 1 hour with

Ammonium Chloride. Sections were then incubated with the primary antibody MAVS/VISA (ThermoFisher a300-782A, 1:250), MAVS (CellSignaling 3993 1:250) and Albimun (Abcam, ab106582 1:500) on 4º overnight, followed by an incubation with the secondary antibodies for 1h at room temperature (1:500). Then, samples were washed with PBS and mounted with aqueous mounting medium with DAPI (1:1000). In this technique, up to 4 representative microphotographs of each human sample was taken with a Thunder Imager tissue microscope (Leica Microsystems). Leica Las X 3.7.4 software was used for acquisition and analysis of immunohistochemistry/immunofluorescence staining.

Immunohistochemistry. For Ki-67, cleaved caspase 3 and collagen type-I, immunohistochemistry staining, samples fixated in paraffin were dewaxed, hydrated, pre-treated in PTLink TE buffer pH 9 and blocked with 3% peroxidase for 10 min. Sections were then incubated with the primary antibody (dakoM7248, DAKO, 1:500) (9664, Cell Signalling, 1:100) overnight and at room temperature, followed by an incubation with the secondary antibody (EnVision, DAKO) for 30 min at room temperature. After that, DAB developer was used for 1 min and sections were counterstained with Mayer´s hematoxylin for 10 min, dehydrated and mounted.

Serum levels of metabolites.

Whole trunk blood obtained during the sacrifice of mice was collected and then spun for 15 min at 6000xg and 4ºC. The supernatant was transferred to a new tube to obtain the serum. Serum ALT activity (41283, Spinreact), AST activity (41273, Spinreact), triglycerides levels (1001310, Spinreact) and total cholesterol (1001093, Spinreact) were

measured by spectrophotometry in a ThermoScientificMultiskan GO spectrophotometer, according to the manufacturer's instructions.

Human cell cultures.

THLE2 human hepatic cell line (American Type Culture Collection, ATCC) was cultured in bronchial epithelial cell basal medium (BEBM) supplemented with a growth factors BulleKit (Lonza/Clonetics Corporation), 70 ng/mL phosphoethanolamine, 5 ng/mL epidermal growth factor, 10% (v/v) FBS and 1% (v/v) Glutamine-Penicillin-Streptomycin solution (MERCK). THLE-2 cells were grown on culture plates pre-coated with a mixture of 0.01 mg/ml fibronectin (#33010018, Sigma Aldrich, USA), 0.01 mg/ml bovine serum albumin (#A4503, Sigma Aldrich, USA) and 0.03 mg/ml collagen type I (#sc-136157, Santa Cruz, USA). For the HepG2 human hepatoma cell line (European Collection of Animal Cell Cultures) we used Minimum Essential Medium Eagle (EMEM) supplemented with 10% (v/v) FBS, 1% (v/v) Glutamine-Penicillin-Streptomycin solution and 1% (v/v) Non-Essential Aminoacids (NEAA, MERCK) as the growth medium. For experiments, HepG2 cells were seeded in pre-coated plates with collagen type I diluted in PBS (1:40). All the cell lines were maintained at 37ºC in a humidified atmosphere containing 5% CO_2.

Isolation and culture of primary hepatocytes.

Primary hepatocytes were isolated from male C57BL/6 WT mice via collagenase perfusion. In brief, animals were anesthetized with isoflurane, the abdomen was opened and a catheter was inserted into the inferior vena cava while the portal vein was cut. Next, liver was washed by perfusion with Krebs-Henseleit (KH) perfusion medium at 37 °C. After the washing, EGTA 0.05% (w/v) was added to the KH medium, and the perfusion

was maintained for 5 min. Finally, an enzymatic digestion was performed during 10-12 min with KH perfusion medium supplemented with Ca^{2+} and 50 mg/ml collagenase type I (LS004196, Worthington). After perfusion, the liver was gently disaggregated. The viable cells were purified by density centrifugation at 500xrpm for 5 min 3 times at 4 °C. Isolated pure hepatocytes were seeded over collagen-coated culture dishes at a density of 4 x 10^5 cells/well in the medium for cell adhesion (serum-free Dulbecco's modified Eagle's medium (DMEM), supplemented with 10% (v/v) FBS, 1% (v/v) Glutamine-Penicillin-Streptomycin solution and 1% (v/v).

MAVS silencing and overexpression.

1.5 x 10^5 THLE2, 3.0 x 10^5 HepG2 or 3.0 x 10^5 primary hepatocytes cells were seeded in six-well plates for the experiments. All cells were transfected with specific small-interference RNA (siRNA) to knockdown the expression of MAVS (siGENOME SMART Pool, Dharmacon). The control group was administered with a non-targeting siRNA (siGENOME Non-targeting siRNA Pool, Dhamacon). The transfection was performed using Dharmafect 1 reagent (Dharmacon) for THLE2 cells and primary hepatocytes, TransIT-siQUEST reagent (Mirus) for HepG2 cells: 0.05 nmol of each siRNA diluted in 250µl of optiMEM (Life Technologies) was mixed with 6.5 µl for THLE2 and 8 µl for primary hepatocytes of Dharmafect 1 (or 7.5 µl of TransIT siQUEST for HepG2) diluted in 243.5 µl of optiMEM; 500 µl of the transfection mixture was added into each well. For plasmid transfection, we used a DNA plasmid containing the sequence necessary to increase the expression of TAp63 (pTAp63 #27008, Addgene), MAVS (pMAVS WT, VB230308-1284kqm, Vector Builder; pMAVS MUT Thr373, VB230308-1284kqm*, Vector Builder) and an empty plasmid as control. For THLE2 and HepG2 cells we used Lipofectamine 2000: 4 µl (or 6 µl for HepG2 cells) Lipofectamine 2000

diluted in 250 µl of optiMEM mixed with 2.5 µg of DNA (MAVS, TAp63α or empty control) diluted on 250 µl of optiMEM. Next, this mixture was added into each well and incubated for 6 h. The medium was replaced with fresh medium after 6 h. For primary hepatocytes we used Lipofectamine 3000: 5 µl Lipofectamine 3000 diluted in 125 µl of optiMEM mixed with 5 µg of DNA (MAVS or empty control) diluted on 125 µl of optiMEM. Next, this mixture was added into each well and incubated overnight. The medium was replaced with fresh medium after overnight incubation. Cells were collected after a total of 24 and/or 48 h to check the efficiency of silencing or overexpression by Real-Time PCR.

Mouse hepatic spheroids.

AML12 hepatocytes were thawed in DMEM high glucose (10565018, ThermoFisher)/Ham's F-12 with l-glutamine (Lonza) and supplemented with 10% FBS, 15 mM HEPES (Carl Roth), 40 ng ml−1 dexamethasone (Sigma Aldrich), 0.005 mg ml−1 (850 nM) human recombinant insulin (Sigma Aldrich), 5 ng ml−1 sodium selenite (Sigma Aldrich) and 0.005 mg ml−1 transferrin (Sigma Aldrich). To create stable lines with the overexpression and silencing of MAVS, 2.5 x 10^5 AML12 cells were seeded in six-well plates. All cells were transfected with the lentiviral MAVS shRNA or control shRNA mixed with polybrene 1ug/ml (TR-1003-G, Millipore), or with DNA plasmid containing the sequence necessary to increase the expression MAVS (pMAVS WT, VB230308-1284kqm, Vector Builder) and an empty plasmid as control. Then, cells were selected by the incubation in complete medium that contained puromycin. One, the cells were selected, they were seeded at the optimized density of 10000 cells per well into round bottom, Pierce 96-well plates (15042, ThermoFisher) in 120 µL volume. Spheroids media was changed every 2–3 days (by removing and adding back 90 µL from 120 µL

media per well). Well-defined spheroids were formed by spontaneous self-aggregation on culture day 5 and maintained until experiments.

Cell treatments.

Primary cultures of hepatocytes from mice overexpressing MAVS were incubated with medium supplemented with 10 µg/ml Adalimumbab (R5010, MERCK), a monoclonal antibody that inhibits TNFα; 10 µM U0126/ HY-12031 (HY-12031, MedChemExpress) a potent and selective ERK1 and ERK2 inhibitor; 8 µg/ml BAY 11-7082 (B5556, Sigma); (a nuclear factor-kappa B (NF-kB) inhibitor; 75 µM PUGNAc (A7229, Sigma), an inhibitor of O-GlcNAc-β-N-acetylglucosaminidase; and 100 µM OSMI-1 (SML1621, Sigma), a cell permeable inhibitor of OGT (O-GlcNAc transferase). These doses were selected after performing a dose- response assay (data not shown). After 24 h, cells were either collected for protein extraction or OR analysis.

Oleic and palmitic acid experiments.

In the study with oleic acid, 0.5 x 10^5 THLE2, 0.7 x 10^5 HepG2, 0.5 x 10^5 primary hepatocytes cells and mouse hepatic spheroids were seeded. Control and MAVS-silenced cells were exposed to FBS-free medium supplemented with 1 mM of oleic acid (MERCK); 0.5-0.25mM of oleic acid and 0.25-0.125mM of palmitic acid, bound to fatty acid-free BSA (Capricorn) at a 2:1 molar ratio for 24 hours to induce lipid accumulation. Controls were supplemented with fatty acid-free BSA alone. The treatment with oleic acid was added after 24 hours of MAVS silencing. Cells were stained with Oil Red O or Biotracker 488 Green Lipid Droplet Dye (SCT144, Millipore) to detect lipids.

Spheroids were photographed in 96 Well Black/Clear Bottom Plate, TC Surface (Thermo Scientific 165305) using the Leica TC-SP5-X-AOBS confocal microscope with white

laser (470–670 nm), equipped with the Leica Application Suite Advanced Fluorescence and using LAS AF software. Was taken serial sections (Z) every 2 µm with 20× objective (HCX PL APO CS 20.0x0.70 DRY UV) and laser line at 500 nm.

Images were collected at resolution 1024 × 1024 pixels and processed for 3D visualization using the maximum intensity projection method. Quantification was performed with imageJ software via ColorDeconvolution2 and the values were corrected by the area of each spheroid.

Mouse TNFα ELISA Kit.

The TNF-α release in cell culture supernatant and in mouse serum was quantified by mouse TNF alpha ELISA Kit (ab208348, Abcam), according to the manufacturer's protocol with intra- and inter-assay coefficients of variation being 6.7% and 9.8%, respectively. Volumes of 50 µL of samples or standard were loaded to appropriate wells. After which, 50 µL of the antibody cocktail were added to each well. The plate was incubated for 1 h at room temperature on a plate shaker set to 400 rpm. The plate was then washed 3 times with 50 µL 1X Wash Buffer PT in each well. Then, 100 µL of TMB development solution were added to each well and incubated for 10 min in the dark on a plate shaker set to 400 rpm. Finally, 100 µL of stop solution in each well were added; the absorbance was read at 450 nm. TNF-α (pg/µL) concentration was calculated by extrapolation of the data in a standard curve constructed with known concentrations of TNF-α. To obtain more accurate data and to exclude misleading results due to eventual differences in the proteins released in the supernatant, data from TNF-α ELISA were normalized by µg of total proteins in each well.

Glucose and Insulin Tolerance Tests (GTT and ITT, respectively).

Basal blood glucose levels were measured after an overnight fast (12 hours) for GTT, and after 6 hours for ITT, with a Glucocard Glucometer (ARKRAY, USA). GTT and ITT were done after an intraperitoneal injection of either 2 g kg−1 D-glucose (G8270, Sigma-Aldrich USA or, 0.35 U kg−1 insulin (Actrapid, Novo Nordisk, Denmark), respectively, and area under curve values were determined.

Real-time PCR.

RNA was extracted from liver and cells samples using TRIzol reagent (Invitrogen) according to the manufacturer's instructions. 100 ng of total RNA were used for each RT reaction, and cDNA synthesis was performed using the SuperScript First-Strand Synthesis System (Invitrogen) and random primers. Negative control reactions, containing all reagents except the sample were used to ensure specificity of the PCR amplification. For Real-time PCR, we used a fluorescent temperature cycler (Applied Biosystems) following the manufacturer's instructions and TaqMan (Applied Biosystems, Life Technologies) for p63 expressions and SYBR green reagent (Agilent Technologies) for other genes. The SYBR green cycling conditions included an initial denaturation at 95ºC for 3 min followed by 40 cycles at 95°C for 5 s and 60°C for 32 s, with a holding stage of 95°C for 15 s, 60ºC for 1 min and 95ºC for 15 s. The TaqMan cycling conditions included an initial denaturation at 50 ºC for 10 min followed by 45 cycles at 95 ºC for 15 s and 60 ºC for 1 min. The specific primers used are described in Supplementary Table 3. All reactions were performed in duplicate using the QuantStudio 5 Real-Time PCR (qPCR). Expression levels were normalized to HPRT1 for each sample and the fold change value was determined from the equation $2^{-\Delta\Delta Ct}$.

Western blot analysis.

Western blots were performed as previously described [6]. Total protein lysates from the liver (20 µg) or cells (6 µg) were subjected to SDS-PAGE, electrotransferred onto polyvinylidene difluoride membranes (BioRad) and probed with the antibodies indicated in Supplementary Table 4.

For the immunoprecipitation assay, extracts were incubated overnight at 4°C with control IgG (m-IgG2a. sc-542731, Santa Cruz Biotechnology) or specific primary antibody (4983s, CellSigna). Antibodies were precipitated with Protein G Agarose beads (Protein G Sepharose 4 Fast Flow. 17-0618-01, GE Healthcare). The captured proteins were centrifuged, the supernatants discarded, and the beads washed in lysis buffer. Beads were boiled for 5 minutes at 95 °C in 20 µl sample buffer. Immunoprecipitated were analyzed by Western blot. The antibodies employed were O-linked N-Acetylglucosamine (2739, Abcam) and MAVS (4983, Cell Signaling and Ab-Bethyl). For protein detection, horseradish peroxidase-conjugated secondary antibodies and chemiluminescence (Amersham Biosciences) were used. Membranes were exposed to radiograph film (Super RX Fuji Medical XRay Film, Fujifilm) and developed with developer and fixing liquids (AGFA) under appropriate dark room conditions. Protein expression was quantified by densitometric analysis with Image J software. Protein levels were normalized to GAPDH and HSP90 for each sample and expressed in relation to the control group.

Two antibodies anti-MAVS were tested since the one from Cell Signaling has been widely used in the literature, and one from Bethyl Labs was used to measure MAVS in the liver of patients with MASLD (5). The anti-MAVS Ab-Bethyl showed a great variability among samples of the same group of patients, with no differences observed in

the quantification of protein levels of MAVS between patients with or without MASLD (Fig. S1A). The specificity of each antibody was tested in HepG2 cells (a human hepatocyte cell line) that had been transfected with siRNA-mediated MAVS or a scrambled snRNA (as a control). Both antibodies showed a marked reduction in MAVS protein levels, even though the efficiency was greater with anti-MAVS Ab-CellSig (Fig. S1B and C). In addition, HepG2 cells were also transfected with an empty plasmid or a plasmid expressing MAVS. While anti-MAVS Ab-CellSig displayed a significant increase in MAVS protein levels, no differences were detected with anti-MAVS Ab-Bethyl (Fig. S1D and E). Taken these results together, we concluded that the specificity of anti-MAVS Ab-CellSig was higher.

In addition, we have performed MAVS staining in sections from livers of people non-MASLD, people with MASLD and people with MASH using the 2 antibodies. Both antibodies seem to be cell-specific, but the Cell Signaling Ab shows an increase in the staining of MAVS in people with MASH, while the Bethyl Ab did not show any difference between the 3 groups (Fig. S2).

Data analysis and statistics.

Data are expressed as mean ± standard error mean (SEM). First, a normality test was performed to test if the populations followed a Gaussian distribution: Kolmogorov–Smirnov test for n between 5 and 7, Shapiro–Wilk test for n ≥ 7. For normal distributions, a parametric test was used: for two population comparisons, an unpaired two-tailed Student´s t-test was used; for multiple comparisons, a one-way analysis of variance (ANOVA) followed by Bonferroni post hoc multiple comparison test was performed. For non-Gaussian distributions, the following were used: Mann–Whitney U tests were used

for two comparison tests and Kruskal–Wallis followed by Dunn post hoc test for multiple comparisons. To study the correlation between gene expression and different parameters, Spearman's (nonparametric) correlation coefficients (r) were used. $p < 0.05$ was considered significant for all the analysis. Analyses were performed with the Prism Software Version 8.0.2 (GraphPad).

REFERENCES

1. Kleiner DE, Brunt EM, Van Natta M, Behling C, Contos MJ, Cummings OW, Ferrell LD, et al. Design and validation of a histological scoring system for nonalcoholic fatty liver disease. Hepatology 2005;41:1313-1321.
2. Gonzalez-Rellan MJ, Parracho T, Heras V, Rodriguez A, Fondevila MF, Novoa E, Lima N, et al. Hepatocyte-specific O-GlcNAc transferase downregulation ameliorates nonalcoholic steatohepatitis by improving mitochondrial function. Mol Metab 2023:101776.
3. da Silva Lima N, Fondevila MF, Novoa E, Buque X, Mercado-Gomez M, Gallet S, Gonzalez-Rellan MJ, et al. Inhibition of ATG3 ameliorates liver steatosis by increasing mitochondrial function. J Hepatol 2022;76:11-24.
4. Covelo-Molares H, Souto Y, Fidalgo M, Guallar D. A Simple, Rapid, and Cost-Effective Method for Loss-of-Function Assays in Pluripotent Cells. Methods Mol Biol 2022;2520:199-213.
5. Fu J, Hu F, Ma T, Zhao WJ, Tian H, Zhang Y, Hu M, et al. A conventional immune regulator mitochondrial antiviral signaling protein blocks hepatic steatosis by maintaining mitochondrial homeostasis. Hepatology 2022;75:403-418.

Figure S1

A

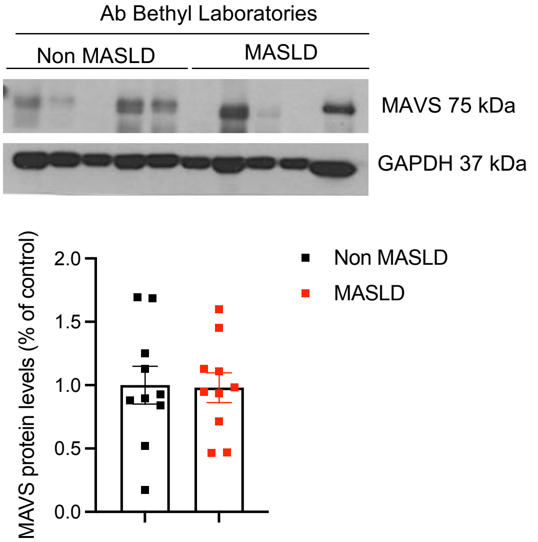

Non MASLD	MASLD
0.927594	1.128927
0.52142	0.468563
0.172646	1.596691
1.691751	0.947526
1.686589	0.467409
1.252083	1.454861
0.878466	0.712159
0.898128	0.984049
0.842396	0.936113
1.128927	1.109079

B

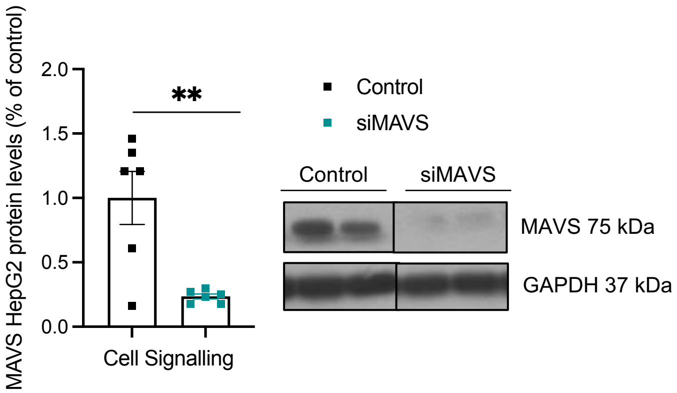

C

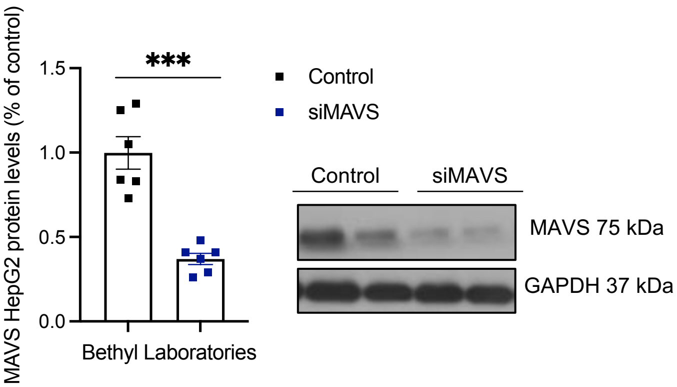

D

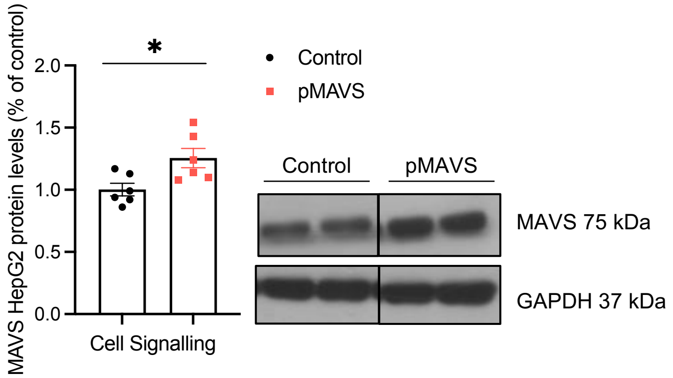

E

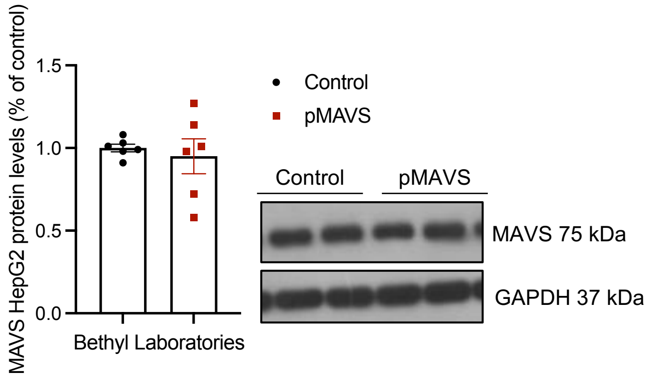

Fig. S1. MAVS protein levels using two different antibodies in human liver and HepG2 cells. (A) MAVS protein levels in the liver of patients without MASLD (non-MASLD) and MASLD (*n* = 10 per group) using the Bethyl Laboratories antibody. (B, C) MAVS protein levels of HepG2 cells with MAVS downregulation using the Cell Signaling antibody or Bethyl Laboratories antibody, respectively (*n* = 6 per group). (D, E) MAVS protein levels of HepG2 cells with MAVS overexpression using the Cell Signaling or Bethyl Laboratories antibody, respectively (*n* = 6 per group). GAPDH was used to normalize protein levels. Data are presented as mean ± SEM; *$p < 0.05$, **$p < 0.01$, ***$p < 0.001$, using a Student's *t*-test.

Figure S2

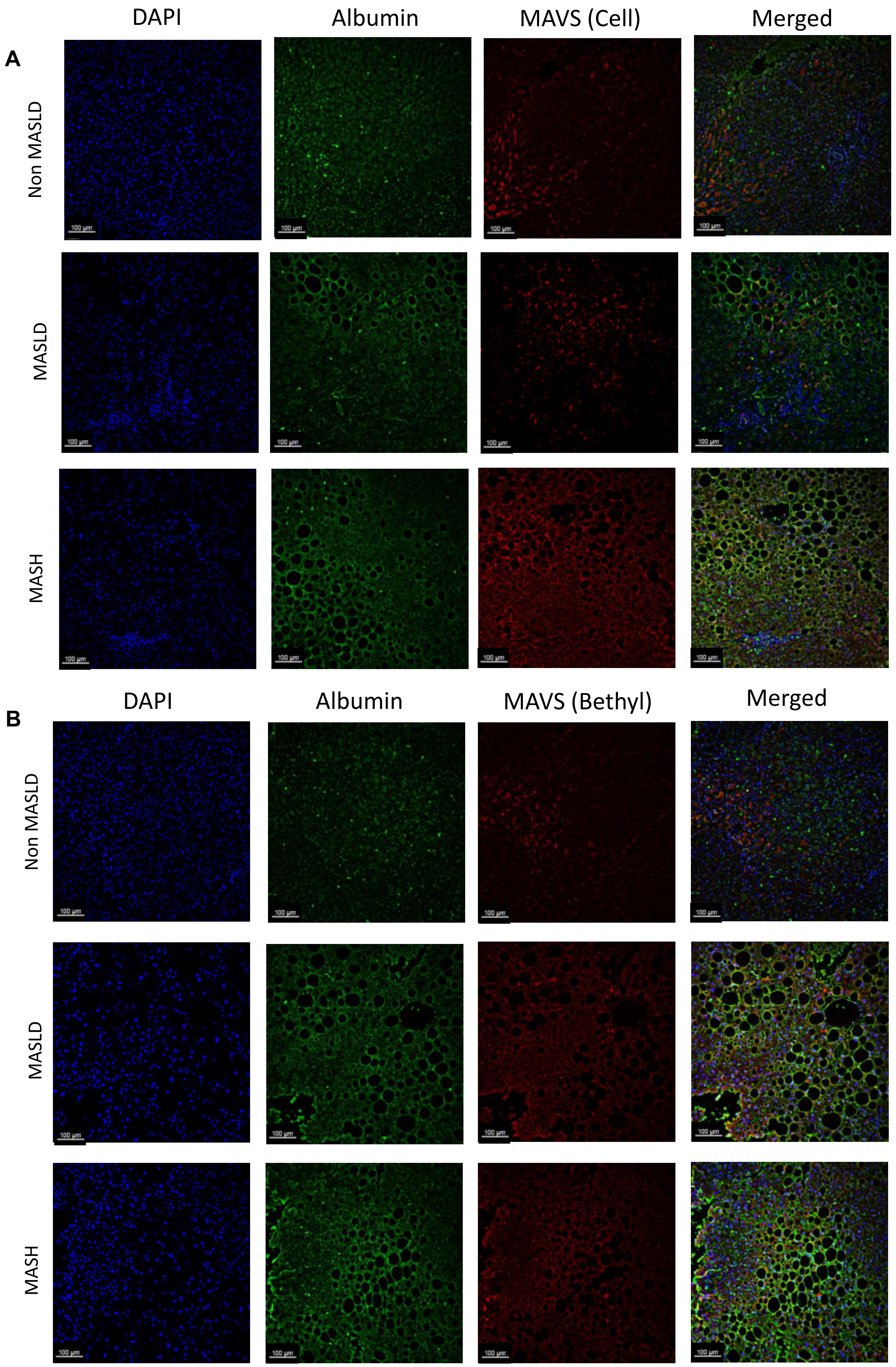
DAPI
Albumin
MAVS (Cell)
Merged
A
Non MASLD
MASLD
MASH
100 µm
B
DAPI
Albumin
MAVS (Bethyl)
Merged
Non MASLD
MASLD
MASH

Fig. S2. Immunofluorescence in human liver from patients with or without MASLD and MASH. (A) Immunofluorescence for DAPI (blue), albumin (green), MAVS (red) and merged in human livers of Non MASLD, MASLD and MASH patients using Cell Signaling antibody. (B) Immunofluorescence for DAPI (blue), albumin (green), MAVS (red) and merged in human livers of Non MASLD, MASLD and MASH patients using Bethyl Laboratories antibody.

Figure S3

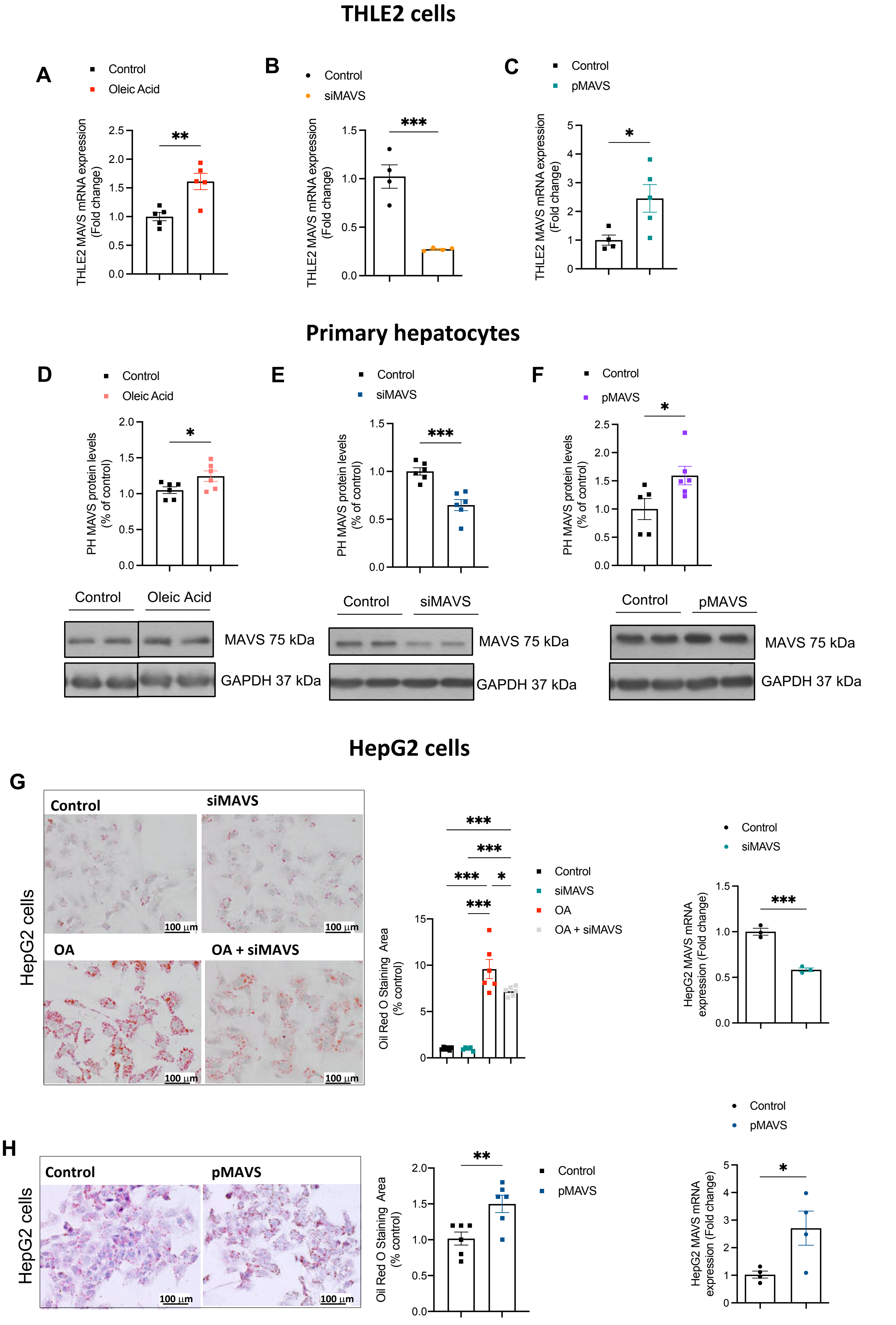

THLE2 cells
A
Control
Oleic Acid
THLE2 MAVS mRNA expression (Fold change)
**
B
Control
siMAVS
THLE2 MAVS mRNA expression (Fold change)
***
C
Control
pMAVS
THLE2 MAVS mRNA expression (Fold change)
*
Primary hepatocytes
D
Control
Oleic Acid
PH MAVS protein levels (% of control)
*
Control
Oleic Acid
MAVS 75 kDa
GAPDH 37 kDa
E
Control
siMAVS
PH MAVS protein levels (% of control)
***
Control
siMAVS
MAVS 75 kDa
GAPDH 37 kDa
F
Control
pMAVS
PH MAVS protein levels (% of control)
*
Control
pMAVS
MAVS 75 kDa
GAPDH 37 kDa
HepG2 cells
G
HepG2 cells
Control
siMAVS
OA
OA + siMAVS
100 µm
Oil Red O Staining Area (% control)
Control
siMAVS
OA
OA + siMAVS
HepG2 MAVS mRNA expression (Fold change)
H
HepG2 cells
Control
pMAVS
100 µm
Oil Red O Staining Area (% control)
**
Control
pMAVS
HepG2 MAVS mRNA expression (Fold change)
*

Fig S3. Protein and mRNA levels of MAVS are modified by different types of treatments. (A) MAVS mRNA expression in THLE2 cells treated with oleic acid (OA) 1mM for 24 h (*n* = 5 per group). (B, C) MAVS mRNA expression in THLE2 cells with MAVS downregulated (siMAVS) or overexpressed (pMAVS), respectively (*n* = 4-5 per group). (D) MAVS protein levels of primary hepatocytes cells treated with OA 1mM for 24 h (*n* = 6 per group). (E, F) MAVS protein levels of primary hepatocytes with MAVS downregulated (siMAVS) or overexpressed (pMAVS), respectively (*n* = 5-6 per group). (G) Representative microphotographs of Oil Red O staining and Oil Red area in HepG2 cells with downregulated MAVS with or without OA 1mM (*n* = 6-8 per group). MAVS mRNA expression in HepG2 cells downregulating MAVS (siMAVS) (*n* = 3 per group). (H) Representative microphotographs of Oil Red O staining and Oil Red Area in HepG2 cells overexpressing MAVS (pMAVS) (*n* = 6 per group). MAVS mRNA levels in HepG2 cells overexpressing MAVS (*n* = 4 per group). HPRT was used to normalize mRNA levels and GAPDH to normalize protein levels. Data are presented as mean ± SEM; *$p < 0.05$, **$p < 0.01$, ***$p < 0.001$, using a Student's *t*-test.

Figure S4

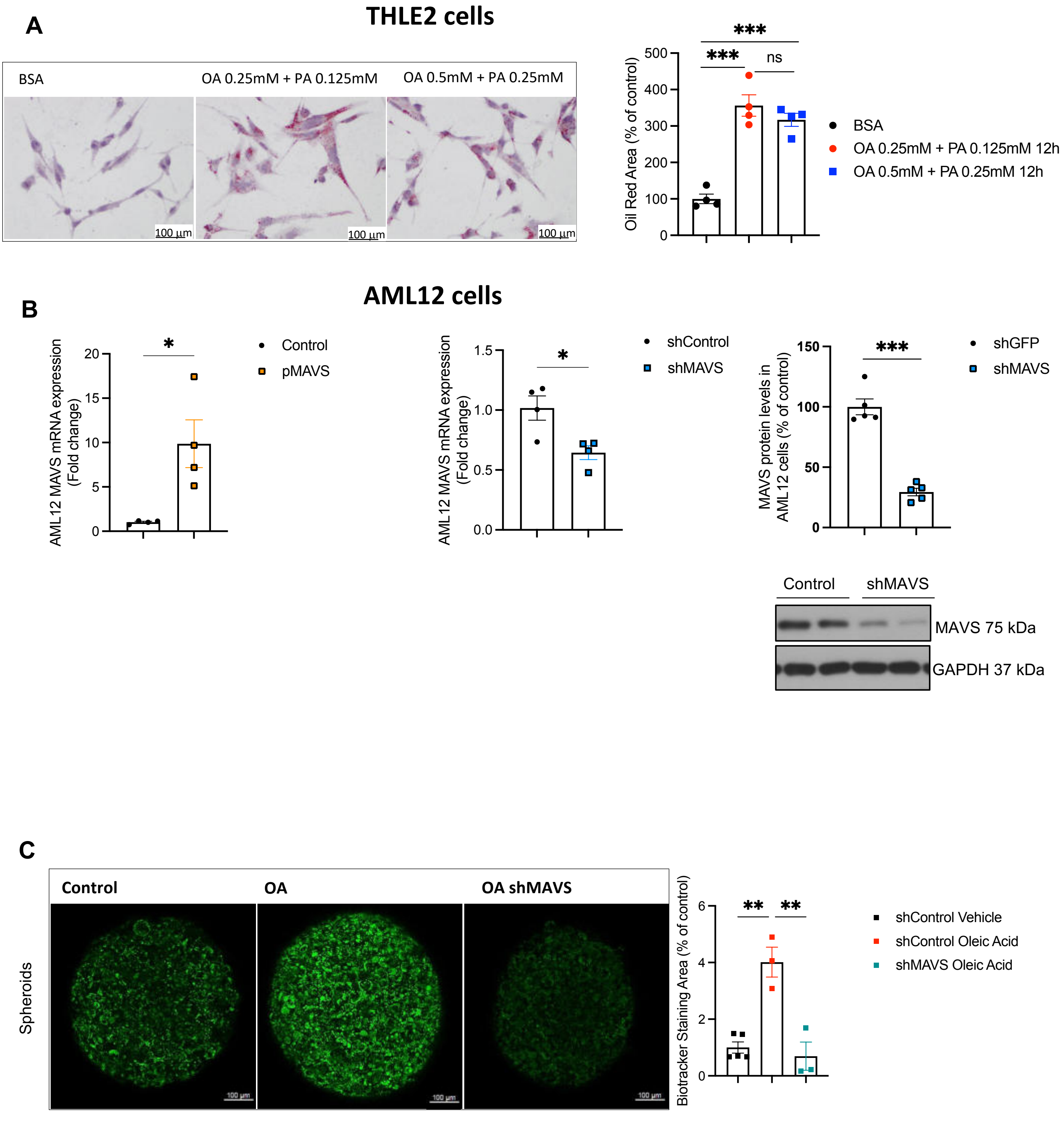
A
THLE2 cells
BSA
OA 0.25mM + PA 0.125mM
OA 0.5mM + PA 0.25mM
100 µm
100 µm
100 µm
Oil Red Area (% of control)
500
400
300
200
100
0
***
***
ns
BSA
OA 0.25mM + PA 0.125mM 12h
OA 0.5mM + PA 0.25mM 12h
B
AML12 cells
AML12 MAVS mRNA expression (Fold change)
20
15
10
5
0
*
Control
pMAVS
AML12 MAVS mRNA expression (Fold change)
1.5
1.0
0.5
0.0
*
shControl
shMAVS
MAVS protein levels in AML 12 cells (% of control)
150
100
50
0
***
shGFP
shMAVS
Control
shMAVS
MAVS 75 kDa
GAPDH 37 kDa
C
Control
OA
OA shMAVS
Spheroids
Biotracker Staining Area (% of control)
6
4
2
0
**
**
shControl Vehicle
shControl Oleic Acid
shMAVS Oleic Acid

Fig. S4. Silencing MAVS inhibits oleate-induced lipid content. (A) Representative microphotographs of Oil Red O staining and Oil Red Area in THLE2 cells treated with different doses of OA 0.25mM and 0.5mM or Palmitate (PA) 0.125mM and 0.25mM (*n* = 4 per group). (B) MAVS mRNA levels in AML12 cells overexpressing MAVS and downregulating MAVS, respectively. MAVS protein levels of AML12 cells downregulating MAVS (*n* = 4-6 per group). (C) Representative microphotographs of Biotracker staining and Biotracker Staining Area of shControl spheroids, shControl spheroids with OA 1mM treatment during 24 h and downregulating MAVS (shMAVS) spheroids with OA treatment during 24 h (*n* = 3-5 per group). HPRT was used to normalize mRNA levels and GAPDH to normalize protein levels. Data are presented as mean ± SEM; $*p < 0.05$, $**p < 0.01$, $***p < 0.001$, using one-way ANOVA followed by a Bonferroni's multiple comparison test (A) and using a Student's *t*-test (B and C).

Figure S5

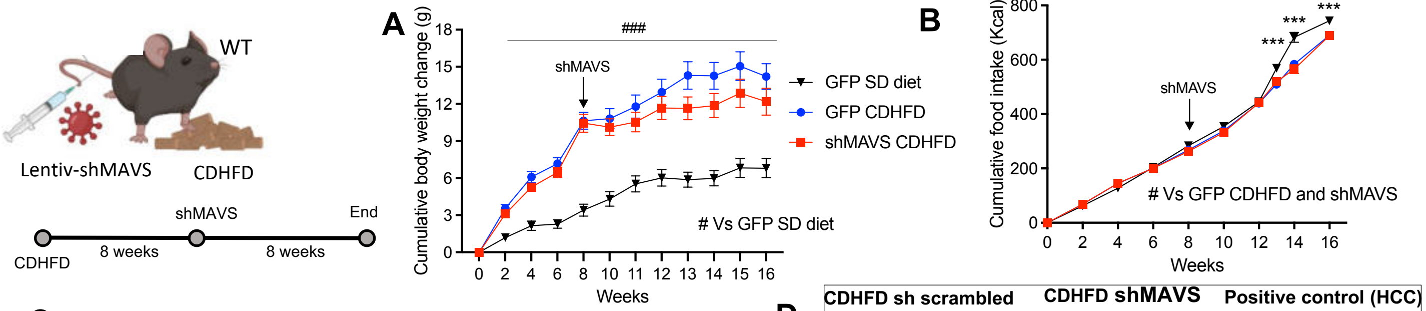

C

	CDHFD sh scrambled (n=10)	CDHFD shMAVS (n=15)
Liver (g/100g of BW)	3.707 ± 0.09	3.612 ± 0.08
Serum triglyceride levels (mg/dl)	93.46 ± 6.95	105.6 ± 5.46
Serum cholesterol levels (mg/dl)	203.5 ± 8.76	194.1 ± 12.58
Serum NEFAs levels (mg/dl)	201.8 ± 21.08	183.2 ± 12.33

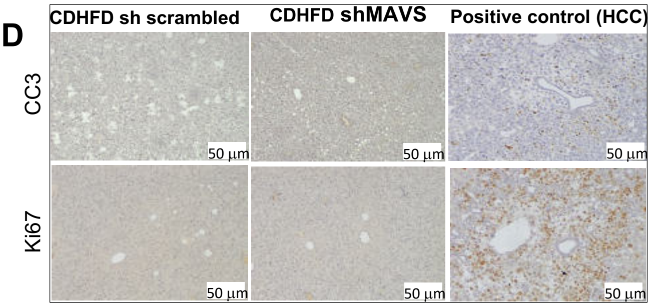

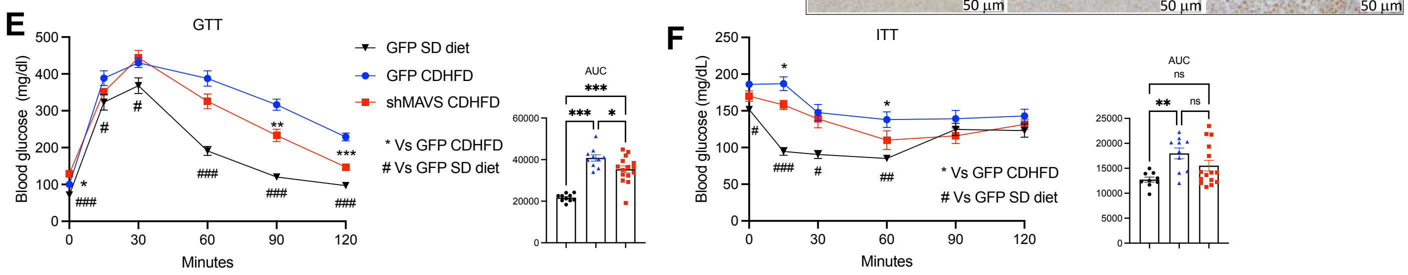

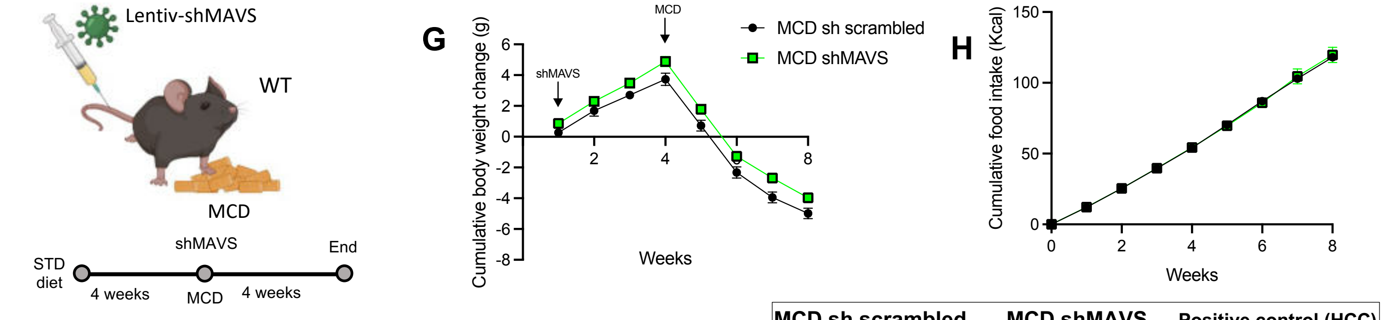

I

	MCD sh scrambled (n=10)	MCD shMAVS (n=10)
Liver (g/100g of BW)	3.949 ± 0.15	4.153 ± 0.05
Serum triglyceride levels (mg/dl)	81.95 ± 2.73	84.51 ± 5.62
Serum cholesterol levels (mg/dl)	49.36 ± 3.33	59.16 ± 4.63

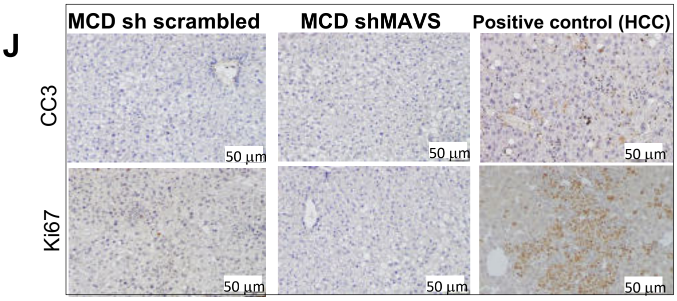

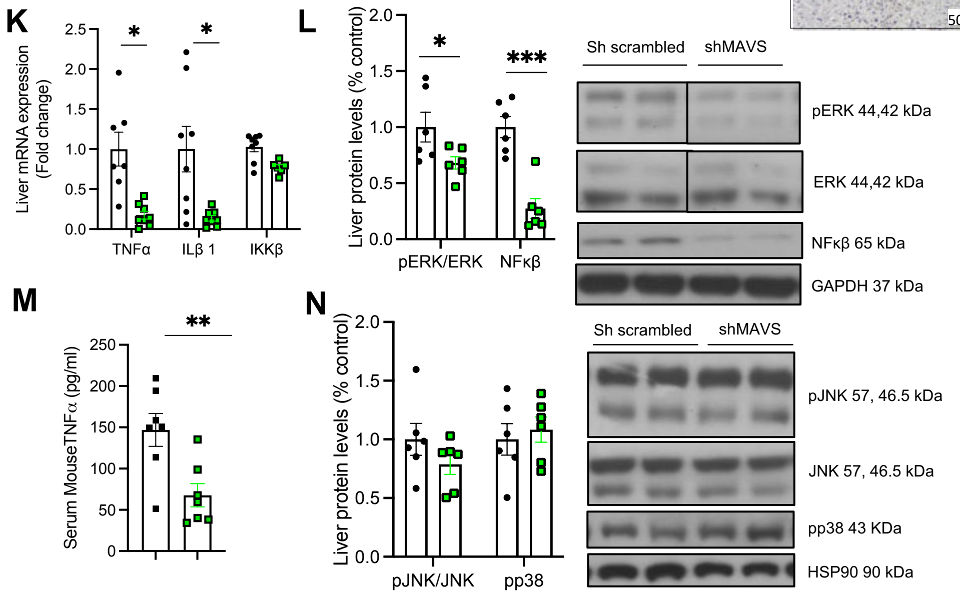

Fig. S5. MAVS downregulation in mice fed CDHFD and MCD diet. (A, B) Cumulative body weight change and cumulative food intake of mice fed a STD diet and CDFHD for 16 weeks, with tail-vein injection (TVI) of a lentivirus encoding shMAVS or shRNA-scrambled control at week 8, as indicated (n = 10–15 per group). (C) Liver mass, serum triglycerides, cholesterol and NEFAs levels of mice fed CDFHD for 16 weeks, with tail-vein injection (TVI) of a lentivirus encoding shMAVS or shRNA-scrambled control at week 8 (D) Liver immunohistochemistry for cleaved caspase 3 (CC3) and Ki67. (E, F) Glucose tolerance test (GTT) and insulin tolerance test (ITT) of mice fed a SD diet and CDFHD for 16 weeks, with tail-vein injection (TVI) of a lentivirus encoding shMAVS or shRNA-scrambled control at week 8, as indicated. (G, H) Cumulative body weight change and cumulative food intake of mice fed a MCD for 4 weeks, with TVI of a lentivirus encoding shMAVS or shRNA-scrambled control after the first week (n = 10 per group). (I) Liver mass, serum triglycerides, cholesterol levels. (J) Liver immunohistochemistry for cleaved caspase 3 (CC3) and Ki67. (K) Liver mRNA expression of inflammatory markers. (L) Liver protein levels of pERK/ERK and NFkβ. (M) Serum levels of TNFα. (N) Liver protein levels of pJNK/JNK and pp38. HPRT was used to normalize mRNA levels. Data with TVI of a lentivirus encoding shMAVS or shRNA-scrambled, as are presented as mean ± SEM; *p < 0.05, **p < 0.01 using one-way ANOVA followed by a Bonferroni's multiple comparison test (A, B, E and F) and Student's t-test (C, D, G, H, I, J, K, L, M and N).

Figure S6

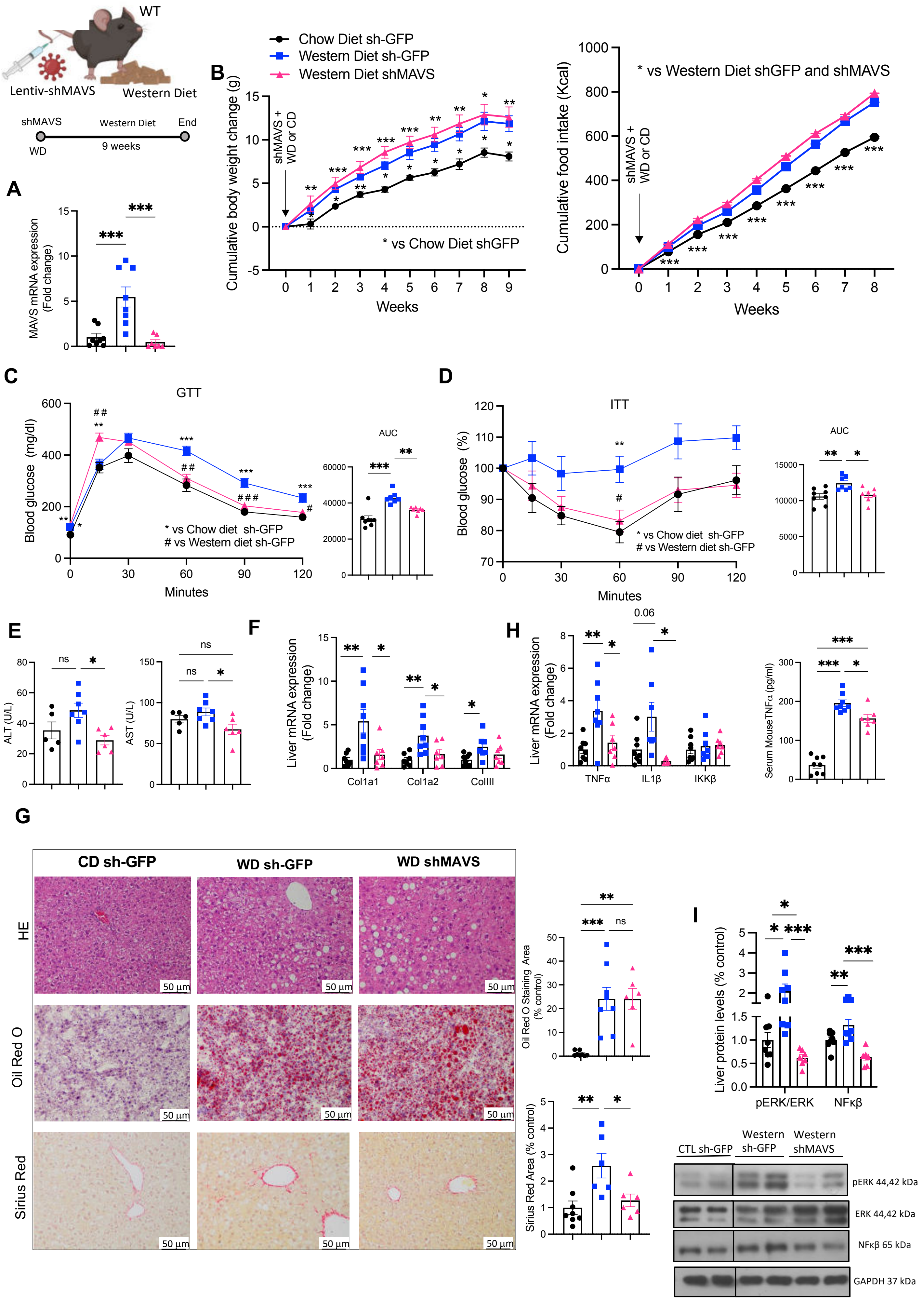
WT
Lentiv-shMAVS
Western Diet
shMAVS
Western Diet
End
WD
9 weeks
A
MAVS mRNA expression (Fold change)
B
Chow Diet sh-GFP
Western Diet sh-GFP
Western Diet shMAVS
shMAVS + WD or CD
Cumulative body weight change (g)
* vs Chow Diet shGFP
Weeks
Cumulative food intake (Kcal)
* vs Western Diet shGFP and shMAVS
Weeks
C
GTT
Blood glucose (mg/dl)
* vs Chow diet sh-GFP
# vs Western diet sh-GFP
Minutes
AUC
D
ITT
Blood glucose (%)
* vs Chow diet sh-GFP
# vs Western diet sh-GFP
Minutes
AUC
E
ALT (U/L)
AST (U/L)
F
Liver mRNA expression (Fold change)
Col1a1
Col1a2
ColIII
H
Liver mRNA expression (Fold change)
TNFα
IL1β
IKKβ
Serum Mouse TNFα (pg/ml)
G
CD sh-GFP
WD sh-GFP
WD shMAVS
HE
Oil Red O
Sirius Red
50 µm
Oil Red O Staining Area (% control)
Sirius Red Area (% control)
I
Liver protein levels (% control)
pERK/ERK
NFκβ
CTL sh-GFP
Western sh-GFP
Western shMAVS
pERK 44,42 kDa
ERK 44,42 kDa
NFκβ 65 kDa
GAPDH 37 kDa

Fig. S6. MAVS downregulation in mice fed Western Diet. (A) Liver mRNA levels of MAVS of mice fed a Chow Diet or Western Diet for 9 weeks, indicated (n= 5–8 per group). (B) Cumulative body weight change and cumulative food intake. (C, D) GTT and ITT, respectively. (E) ALT and AST serum levels. (F) Liver mRNA expression of fibrosis markers. (G) Representative microphotographs of Oil Red O staining and Sirius Red staining. (H) Liver mRNA expression of inflammatory markers and serum TFNα levels. (I) Liver pERK/ERK and NFkβ protein levels. HPRT was used to normalize mRNA levels and GAPDH to normalize protein levels. Data are presented as mean ± SEM; $*p < 0.05$, $**p < 0.01$ using one-way ANOVA followed by a Bonferroni's multiple comparison test.

Figure S7

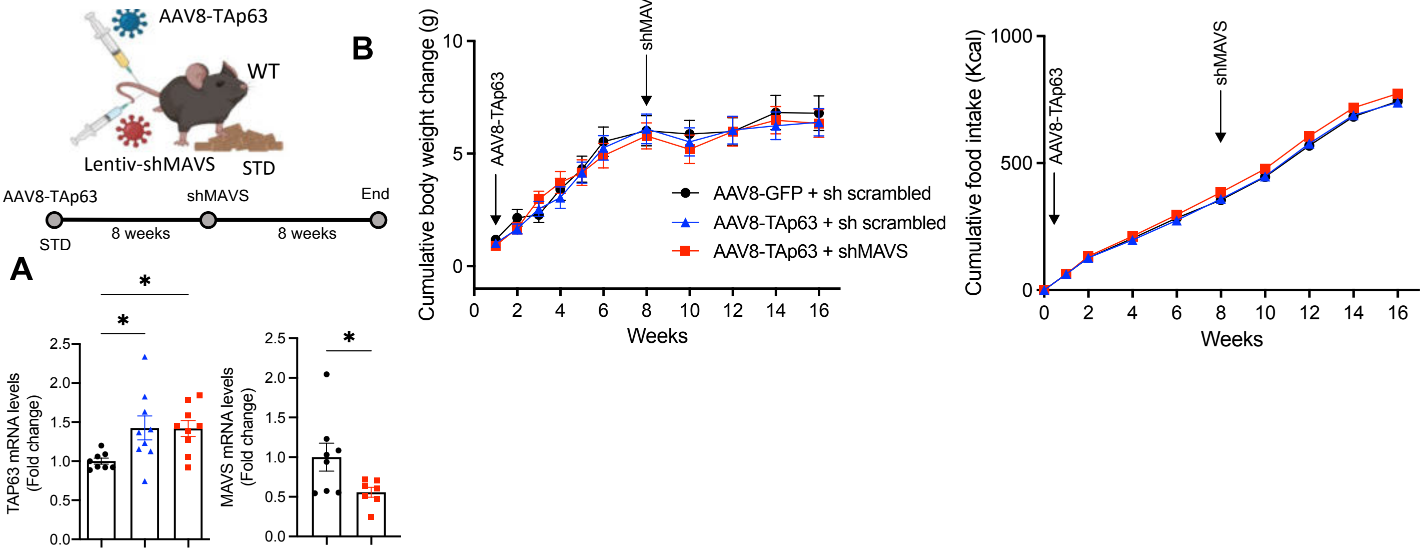

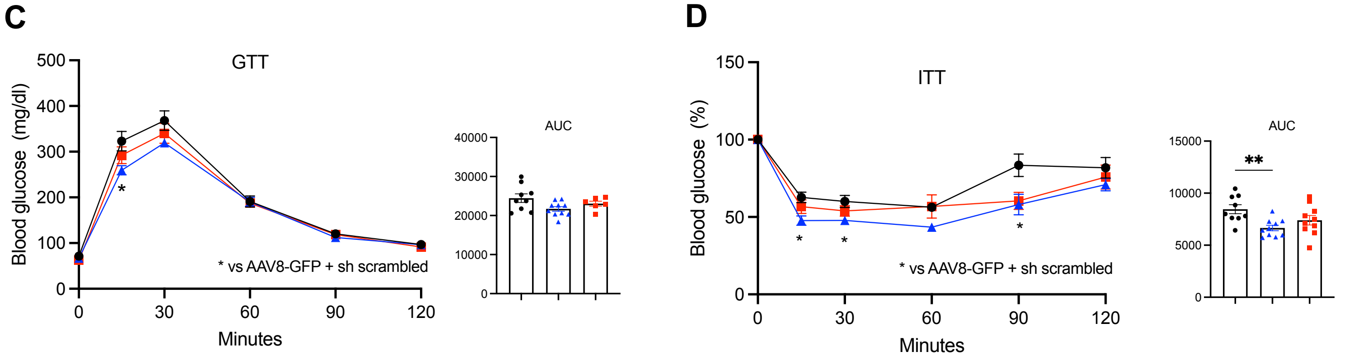

E

	AAV8-GFP + sh scrambled (n=8)	AAV8-TAp63 + sh scrambled (n=10)	AAV8-TAp63 + shMAVS (n=10)
Liver (g/100g of BW)	4.29 ± 0.18	4.23 ± 0.11	4.34 ± 0.09
Serum triglyceride levels (mg/dl)	99.61 ± 7.71	88.41 ± 9.91	90..69 ± 1.81
Serum cholesterol levels (mg/dl)	95.19 ± 4.02	91.11 ± 4.58	102.7 ± 1.99
Serum NEFAs levels (mg/dl)	193.8 ± 18.87	210.3 ± 18.63	104.2 ± 28.69 *

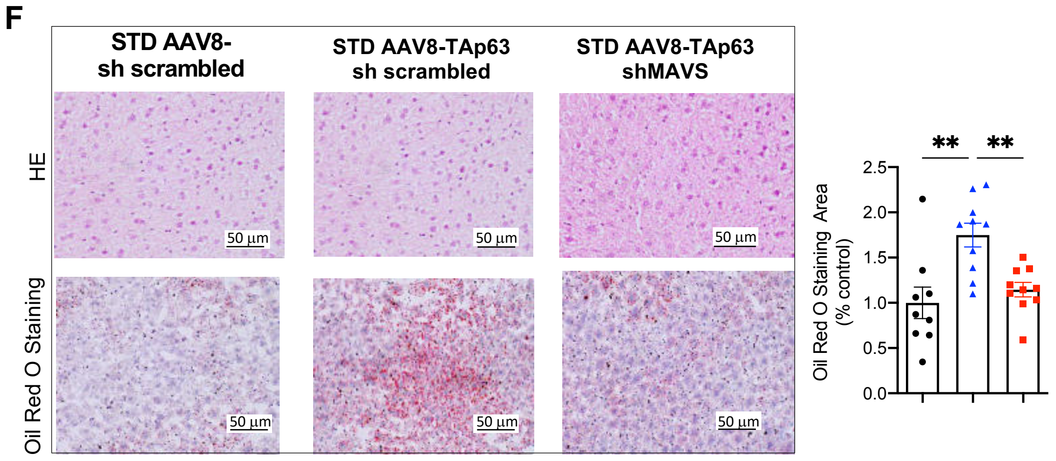

Fig. S7. Liver-specific downregulation of MAVS ameliorated lipid accumulation and fibrosis induced by TAp63α. (A) Hepatic mRNA levels of TAp63α and MAVS. (B) Cumulative body weight change and cumulative food intake of mice fed a standard diet for 16 weeks, with tail-vein injection (TVI) of AAV8–TAp63α or AAV8–GFP after week 1, and a second TVI of lentivirus encoding the shRNA-MAVS or shRNA-scrambled control after week 8, as indicated (n = 8-10 per group). (C, D) GTT and ITT, respectively. (E) Liver mass, serum triglycerides, cholesterol and NEFAs levels. (F) Representative microphotographs of hematoxylin and eosin (upper panel) and Oil Red O staining (lower panel) of liver sections. Oil Red O stain sections (red area) were quantified using ImageJ. HPRT was used to normalize mRNA levels. Data are presented as mean ± SEM; $*p < 0.05$, $**p < 0.01$ using one-way ANOVA followed by a Bonferroni's multiple comparison test.

Figure S8

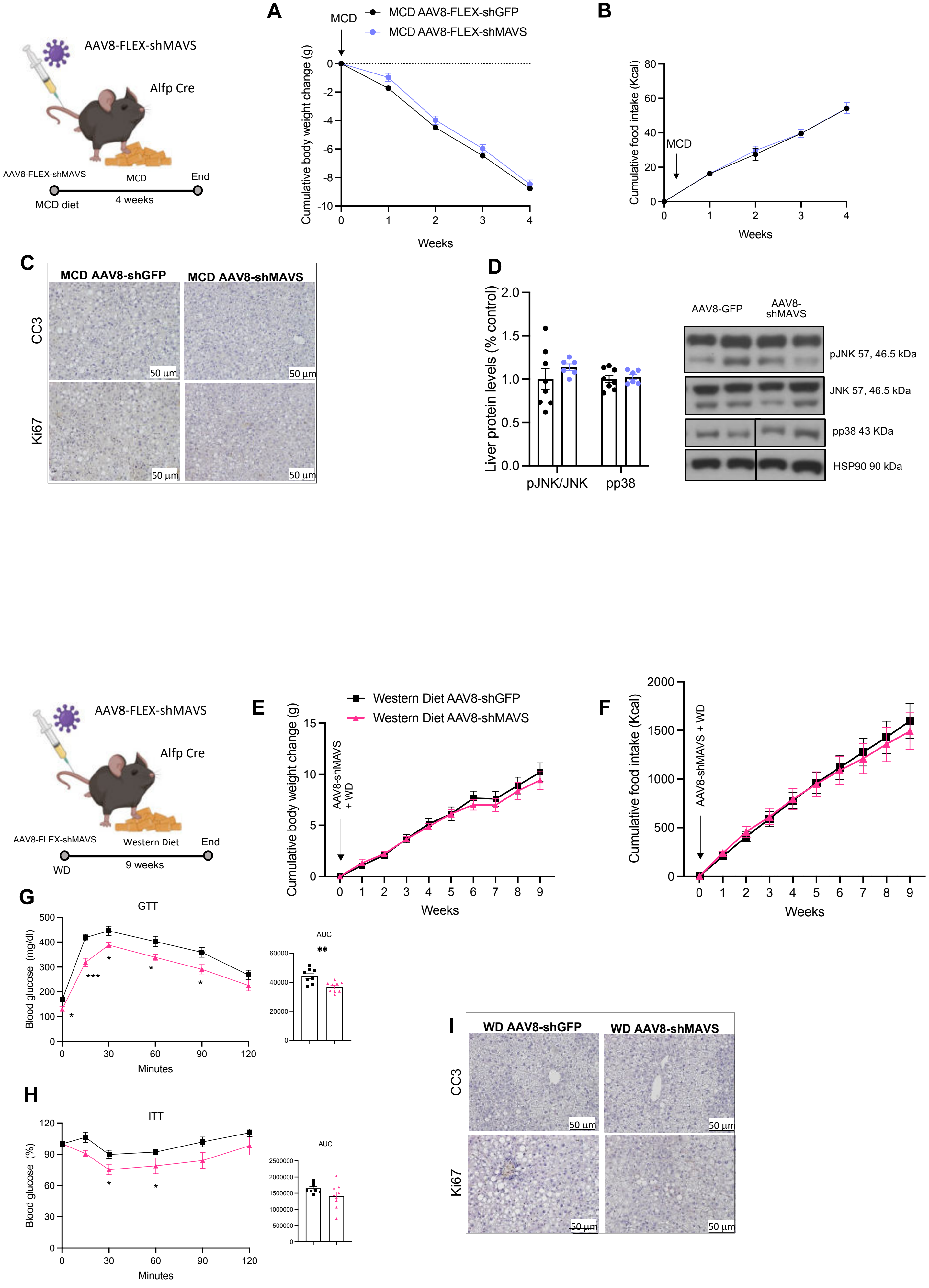

A
B
C
D
E
F
G
H
I
AAV8-FLEX-shMAVS
Alfp Cre
AAV8-FLEX-shMAVS
MCD
End
MCD diet
4 weeks
MCD
MCD AAV8-FLEX-shGFP
MCD AAV8-FLEX-shMAVS
Cumulative body weight change (g)
Weeks
Cumulative food intake (Kcal)
MCD AAV8-shGFP
MCD AAV8-shMAVS
CC3
Ki67
50 µm
Liver protein levels (% control)
pJNK/JNK
pp38
AAV8-GFP
AAV8-shMAVS
pJNK 57, 46.5 kDa
JNK 57, 46.5 kDa
pp38 43 KDa
HSP90 90 kDa
AAV8-FLEX-shMAVS
Western Diet
End
WD
9 weeks
Western Diet AAV8-shGFP
Western Diet AAV8-shMAVS
AAV8-shMAVS + WD
GTT
Blood glucose (mg/dl)
Minutes
AUC
ITT
Blood glucose (%)
WD AAV8-shGFP
WD AAV8-shMAVS

Fig. S8. Hepatocyte-specific downregulation of MAVS in mice fed methionine-and-choline deficient (MCD) diet and Western Diet. (A, B) Cumulative body weight change and cumulative food intake of mice fed a MCD diet for 4 weeks, which received tail vein injection of AAV8-FLEX-shMAVS or AAV8-FLEX-GFP as indicated (n= 6–9 per group). (C) Liver immunohistochemistry for cleaved caspase 3 (CC3) and Ki67. (D) Liver protein levels of pJNK/JNK and pp38. (E, F) Cumulative body weight change and cumulative food intake of mice fed a Western Diet for 9 weeks, which received tail vein injection of AAV8-FLEX-shMAVS or AAV8-FLEX-shGFP, as indicated (n= 11-12 per group). (G, H) GTT and ITT, respectively (I) Liver immunohistochemistry for cleaved caspase 3 (CC3) and Ki67. HPRT was used to normalize mRNA levels and GAPDH to normalize protein levels. Data are presented as mean ± SEM; *$p < 0.05$, **$p < 0.01$, ***$p < 0.001$, using a Student's *t*-test.

Figure S9

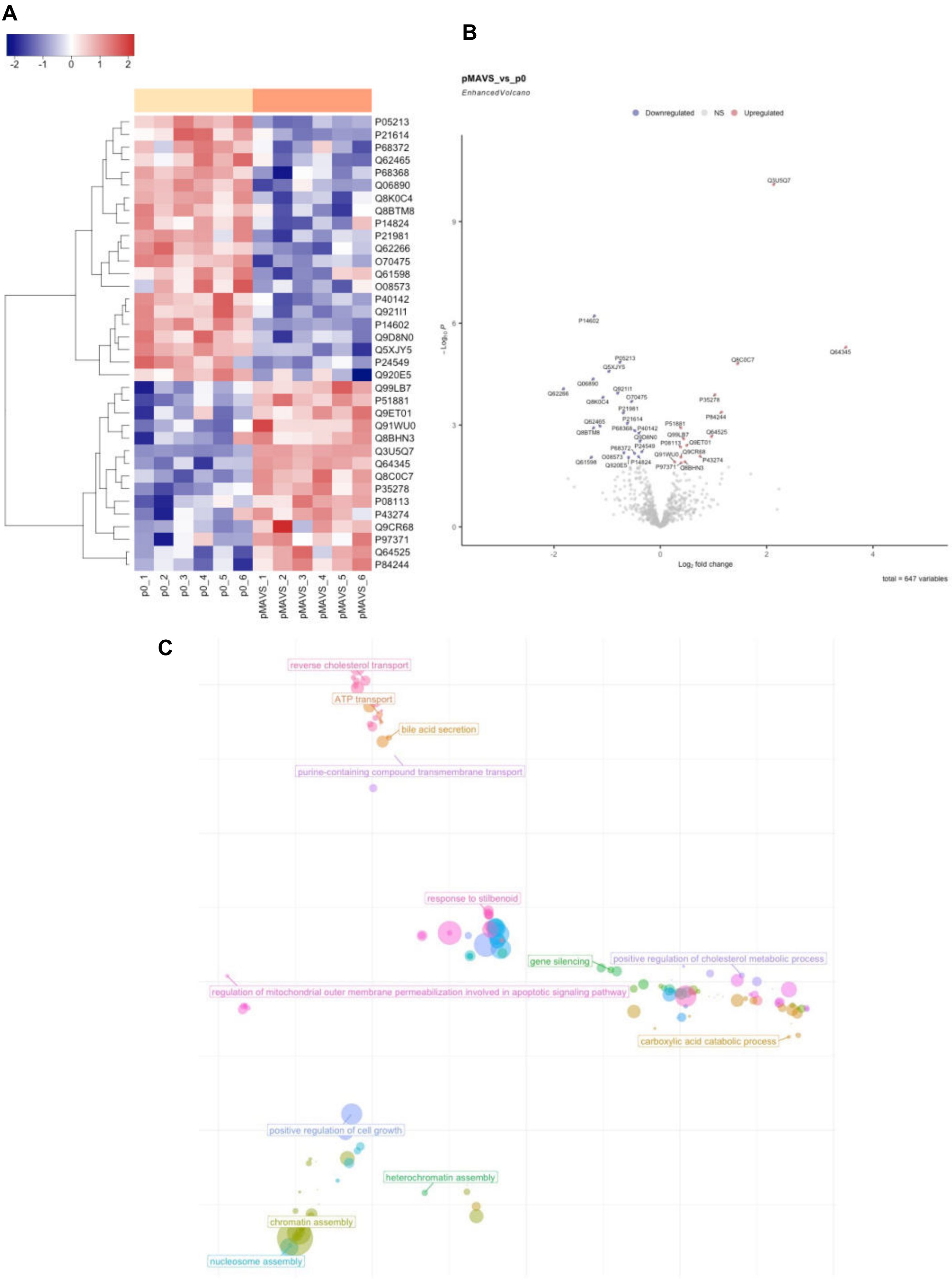
A
-2
-1
0
1
2
P05213
P21614
P68372
Q62465
P68368
Q06890
Q8K0C4
Q8BTM8
P14824
P21981
Q62266
O70475
Q61598
O08573
P40142
Q921I1
P14602
Q9D8N0
Q5XJY5
P24549
Q920E5
Q99LB7
P51881
Q9ET01
Q91WU0
Q8BHN3
Q3U5Q7
Q64345
Q8C0C7
P35278
P08113
P43274
Q9CR68
P97371
Q64525
P84244
p0_1
p0_2
p0_3
p0_4
p0_5
p0_6
pMAVS_1
pMAVS_2
pMAVS_3
pMAVS_4
pMAVS_5
pMAVS_6
B
pMAVS_vs_p0
EnhancedVolcano
Downregulated
NS
Upregulated
Q3U5Q7
P14602
Q64345
Q8C0C7
$-Log_{10}$ P
Log_2 fold change
total = 647 variables
C
reverse cholesterol transport
ATP transport
bile acid secretion
purine-containing compound transmembrane transport
response to stilbenoid
gene silencing
positive regulation of cholesterol metabolic process
regulation of mitochondrial outer membrane permeabilization involved in apoptotic signaling pathway
carboxylic acid catabolic process
positive regulation of cell growth
heterochromatin assembly
chromatin assembly
nucleosome assembly

Fig S9. Proteomic assay in primary hepatocytes with MAVS overexpression. (A) Heatmap, (B) Volcano plot and, (C) Scatter plot of primary hepatocytes with MAVS overexpression. The size of the circumference is proportional to the number of proteins deregulated in the metabolic pathway.

Figure S10

Figure 1E

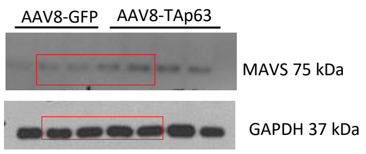

Figure 1F

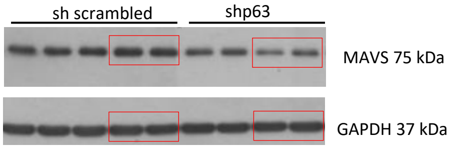

Figure 2B

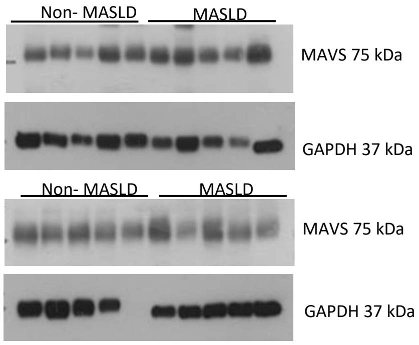

Figure 6A

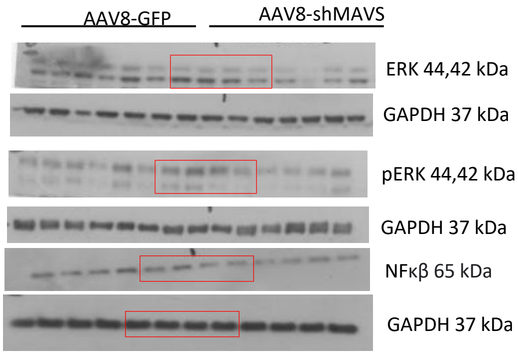

Figure 6C

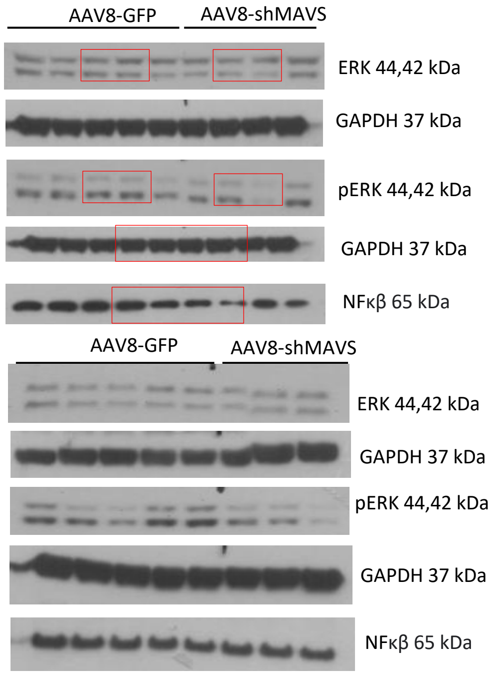

Figure 6E

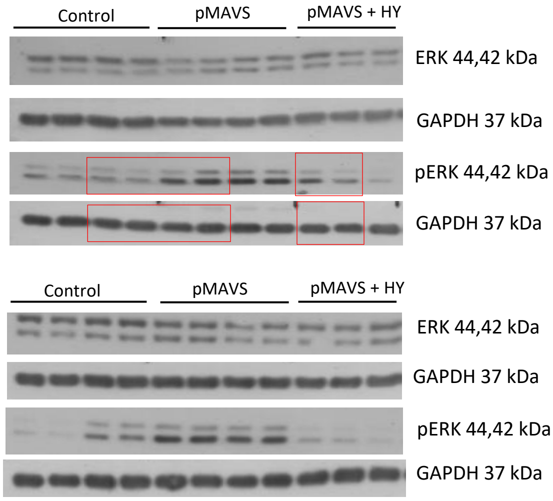

Figure 6K

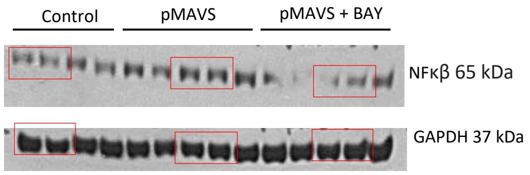

Figure 7A

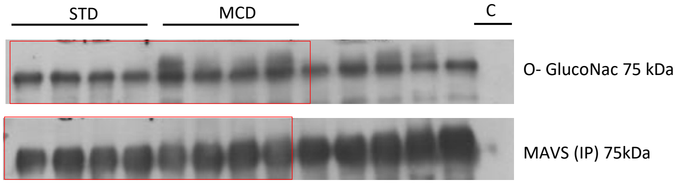

Figure 7C

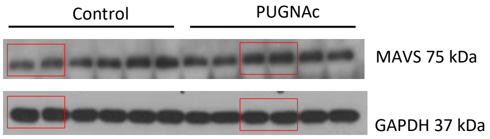

Figure 7D

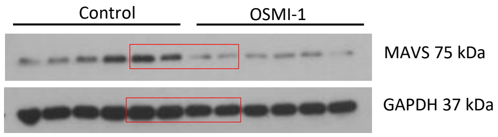

Figure 7G

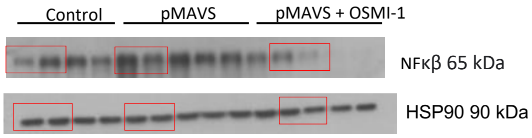

Figure 7J

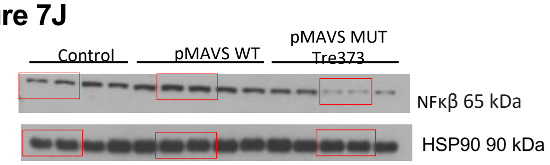

Figure S10

Supplementary Figure 1A

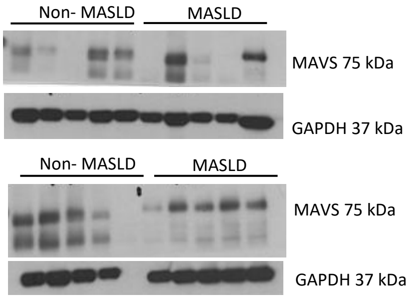

Supplementary Figure 1B

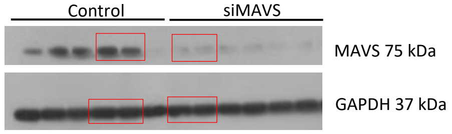

Supplementary Figure 1C

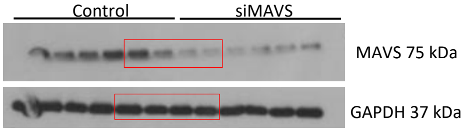

Supplementary Figure 1D

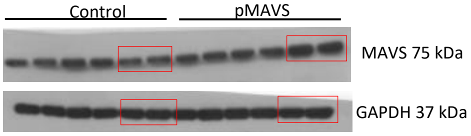

Supplementary Figure 1E

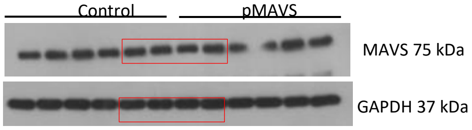

Supplementary Figure 3D

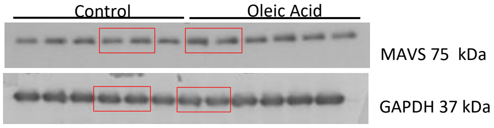

Supplementary Figure 3E

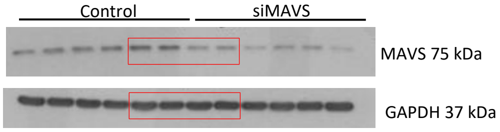

Supplementary Figure 3F

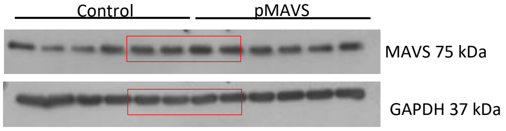

Supplementary Figure 4B

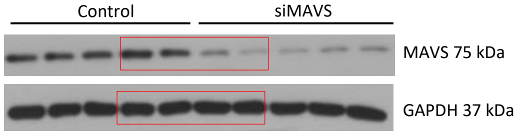

Supplementary Figure 5L

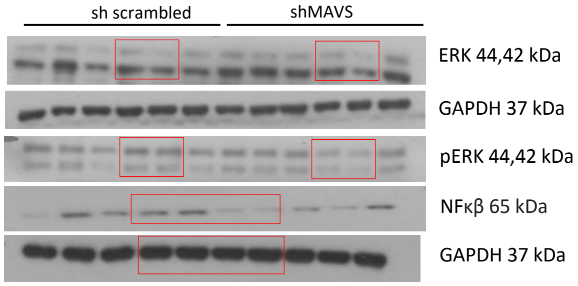

Supplementary Figure 5N

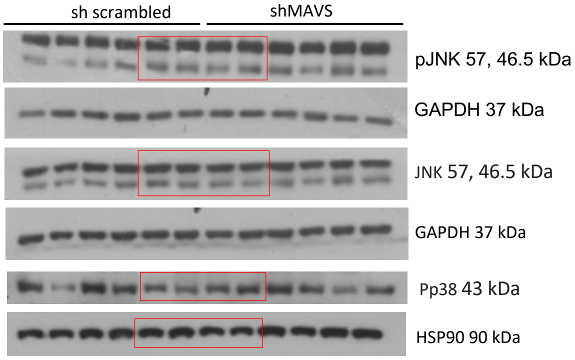

Supplementary Figure 6I

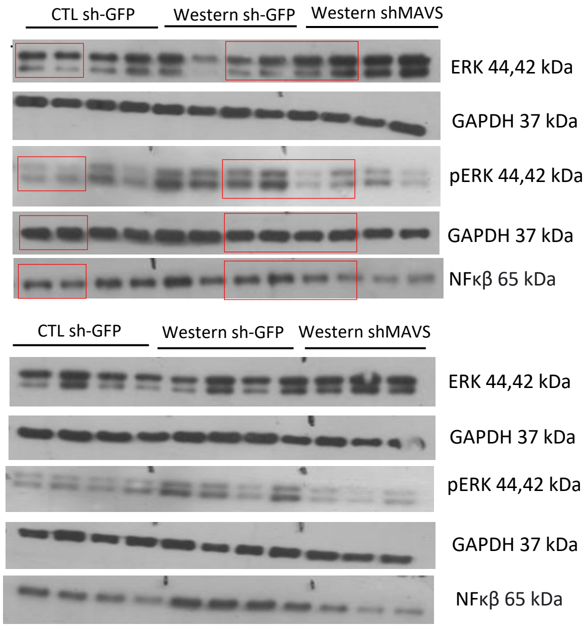

Supplementary Figure 8D

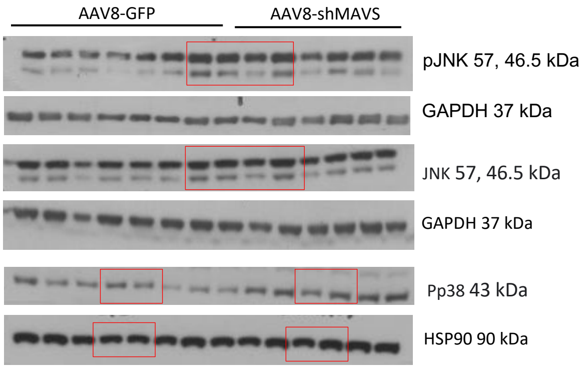

Fig S10. Uncropped western blot analyzed in this study.

Supplementaty Table 1. Macronutrients composition of rodent diets used.

**Rodent Diets**	**Standard Diet** (Inotiv, Teklad Global)		**CDHFD** (Research Diet, D05010402)		**MCD** (Research Diet, A02082002BR)		**Chow Diet** (Ssniff, TD. 88137)		**Western Diet** (Ssniff, CD. 88137)	
	**gm%**	**kcal %**	**gm %**	**kcal %**	**gm%**	**kcal %**	**gm %**	**kcal %**	**gm%**	**kcal %**
**Protein**	18.4	24	23.7	20	17	16	17.5	18	17.3	15
**Carbohydrates**	44.2 (avaliable)	58	41.5	35.1	66	63	64.7	69	48.7	43
**Fat**	6	18	23.7	44.9	10	21	5.1	13	21.1	42
**Total**		100		100		100		100		
**Kcal/gm**	3.1		4.74		4.2		3.75		4.575	100

Supplementary table 2: Anthropometric, biochemical and clinical characteristics of patients with normal liver (Non MASLD) and metabolic dysfunction-associated steatotic liver disease (MASLD) (n=24). Data are shown as mean ± standard deviation (SD) or as number of cases (%). ALT, alanine aminotransferase; AST, aspartate amino transferase; LDL, low-density lipoprotein.

**Feature**	**Non MASLD (n= 12)**	**MASLD (n = 11)**
Age (years)	48.0 ± 7.1	48.45 ± 11.7
Body mass index (kg/m^2)	39.9 ± 6.4	41.9 ± 7.6
Sex (%)		
Men	8 (66%)	6 (54%)
Women	4 (34%)	5 (46%)
Body weight (kg)	118.1 ± 26.3	117.0 ± 22.2
Glucose (mg/dL)	110.4 ± 54.7	133 ± 66.7
LDL cholesterol (mg/dL)	115.5 ± 34.7	108.8 ± 35.5
Triglycerides (mg/dL)	112.7 ± 55.2	181.7± 106.8
Total cholesterol (mg/dL)	191.3 ± 42.1	192.6 ± 50.0
ALT (IU/L)	28.8 ± 8.7	36.8 ± 17.4
AST (IU/L)	19.8 ± 9.6	23.8 ± 10.22
NAS Score (%)		
Grade 0	34	0
Grade 1	58	0
Grade 2	8	0
Grade 3	0	36
Grade 4	0	9
Grade 5	0	46
Grade 6	0	9
Fibrosis (%)		
Stage 0	92	8
Stage 1	8	36
Stage 2	0	56

Supplementary table 3: List of primers used.

	Forward (5'-3')	Reverse (5'-3')
**MAVS Mm**	ATCAGGAGCTTGCACTCTGG	GGAGCTGCTAGGGCTAATGG
**MAVS Hs**	GTGGAGTACTTCATTGCGGC	CTCAGCAGGAAGTGACGGTG
**TNFα Mm**	AGCCCCCAGTCTGTATCCTT	CTCCCTTTGCAGAACTCAGG
**IL1β Mm**	ACTCATTGTGGCTGTGGAGA	TTGTTCATCTCGGAGCCTGT
**Col1α1 Mm**	CCTAATGCTGCCTTTTCTGC	ATGTCCCAGCAGGATTTGAG
**Col1α2 Mm**	CCGTGCTTCTCAGAACATCA	CTTGCCCCATTCATTTGTCT
**Col III Mm**	GCACAGCAGTCCAACGTA	TCTCCAAATGGGATCTCT
**IKKβ Mm**	TGCAGGACACTGTGAAGGAG	CTGGCAGAGTGAGATGTCCA
**HPRT Mm**	AAGCTTGCTGGTGAAAAGGA	TTGCGCTCATCTTAGGCTTT
**HPRT Hs**	ACCCCACGAAGTGTTGGATA	AAGCAGATGGCCACAGAACT
**HPRT Hs**	TaqMan: Hs02800695_m1	
**TAp63 Mm**	TaqMan: Mm00495793_m1	

Supplementary table 4: Antibodies

Antibody	Reference	Host	Dilution
**MAVS Human**	Bettyl Laboratories (A300-782A)	Rabbit polyclonal	1:1000
**MAVS Rodent**	Cell Signaling (4983)	Rabbit monoclonal	1:1000
**MAVS Human**	Cell Signaling (3993)	Rabbit polyclonal	1:1000
**NFκβ**	Santa Cruz (SC372)	Rabbit polyclonal	1:1000
**O linked N-Acetylglucosamine**	Abcam (AB2739)	Mouse monoclonal	1:1000
**Glyceraldehyde 3-phosphatedehydrogenase, GAPDH**	Merck (CB1001)	Mouse monoclonal	1:1000
**SAPK/JNK**	Cell Signaling (9252)	Rabbit polyclonal	1:1000
**Phospho-SAPK/JNK**	Cell Signaling (9251)	Rabbit polyclonal	1:1000
**Phospho-p38 MAPK**	Cell Signaling (4511)	Rabbit polyclonal	1:1000
**P44/42 MAPK (Erk1/2)**	Cell Signaling (9102)	Rabbit polyclonal	1:1000
**Phospho-p44/42 MAPK (Erk1/2)**	Cell Signaling (9101)	Rabbit polyclonal	1:1000
**HSP90**	Santa Cruz (13119)	Mouse monoclonal	1:1000
